# Optimal dosage ranges of various exercise types for enhancing timed up and go performance in Parkinson’s disease patients: a systematic review and Bayesian network meta-analysis

**DOI:** 10.3389/fnagi.2024.1399175

**Published:** 2024-06-24

**Authors:** Yuan Yuan, JunYu Wang, GuoTuan Wang, Tao Wang, HaoYang Zhang, XueYing Fu, LiHua Wu, XiaoTian Chen, Rui Xia, Lin Zhang, Shu-Cheng Lin, Yong Yang

**Affiliations:** ^1^Department of Physical Education, Kunsan National University, Gunsan-si, Jeollabuk-do, Republic of Korea; ^2^The School of Exercise and Health, Shanghai University of Sport, Shanghai, China; ^3^Institute of Physical Education, Henan University, Kaifeng, China; ^4^School of Physical Education and Health, Krasnoyarsk State Pedagogical University named after V.P., Krasnoyarsk City, Russia; ^5^College of Physical Education and Health, Southwest University of Science and Technology, Mianyang, China; ^6^The Seventh Clinical Medical College, Guangzhou University of Chinese Medicine, Guangzhou, China; ^7^Second Clinical Medical College, Wenzhou Medical University, Zhejiang, China; ^8^Laboratory of Kinesiology and Rehabilitation, School of Physical Education and Sport, Chaohu University, Hefei, China; ^9^Department of Rehabilitation, West China Hospital Sichuan University Jintang Hospital, Chengdu, China; ^10^Department of Sport, Leisure and Health Management, Tainan University of Technology, Tainan City, Taiwan

**Keywords:** Parkinson’s disease, exercise, Timed and up go, dose–response, RCTs, Bayesian network meta-analysis

## Abstract

**Objective:**

To examine the dose–response relationship between specific types of exercise for alleviating Timed up and Go (TUG) in Parkinson’s disease PD.

**Design:**

Systematic review and Bayesian network meta-analysis.

**Data sources:**

PubMed, Medline, Embase, PsycINFO, Cochrane Library, and Web of Science were searched from inception until February 5th, 2024.

**Study analysis:**

Data analysis was conducted using R software with the MBNMA package. Effect sizes of outcome indicators were expressed as mean deviation (MD) and 95% confidence intervals (95% CrI). The risk of bias in the network was evaluated independently by two reviewers using ROB2.

**Results:**

A total of 73 studies involving 3,354 PD patients. The text discusses dose–response relationships in improving TUG performance among PD patients across various exercise types. Notably, Aquatic (AQE), Mix Exercise (Mul_C), Sensory Exercise (SE), and Resistance Training (RT) demonstrate effective dose ranges, with AQE optimal at 1500 METs-min/week (MD: −8.359, 95% CI: −1.398 to −2.648), Mul_C at 1000 METs-min/week (MD: −4.551, 95% CI: −8.083 to −0.946), SE at 1200 METs-min/week (MD: −5.145, 95% CI: −9.643 to −0.472), and RT at 610 METs-min/week (MD: −2.187, 95% CI: −3.161 to −1.278), respectively. However, no effective doses are found for Aerobic Exercise (AE), Balance Gait Training (BGT), Dance, and Treadmill Training (TT). Mind–body exercise (MBE) shows promise with an effective range of 130 to 750 METs-min/week and an optimal dose of 750 METs-min/week (MD: −2.822, 95% CI: −4.604 to −0.996). According to the GRADE system, the included studies’ overall quality of the evidence was identified moderate level.

**Conclusion:**

This study identifies specific exercise modalities and dosages that significantly enhance TUG performance in PD patients. AQE emerges as the most effective modality, with an optimal dosage of 1,500 METs-min/week. MBE shows significant benefits at lower dosages, catering to patients with varying exercise capacities. RT exhibits a nuanced “U-shaped” dose–response relationship, suggesting an optimal range balancing efficacy and the risk of overtraining. These findings advocate for tailored exercise programs in PD management, emphasizing personalized prescriptions to maximize outcomes.

**Systematic Review Registration**: International Prospective Register of Systematic Reviews (PROSPERO) (CRD42024506968).

## Introduction

TUG (Timed up-and-go test) has been widely accepted as a standard assessment for measuring the basic motor symptoms of PD and has been widely accepted in clinical practices for over 20 years (Yoo et al., 2020). A cross-sectional cohort study demonstrated that the TUG is an accurate assessment tool for identifying those with PD who are at risk for falls (Nocera et al., 2013). In a TUG test several basic mobility sub-tasks included “Sit,” “Sit-to-Stand,” “Walk,” “Turn,” “Walk-Back,” and “Sit-Back” (Li et al., 2018). Because the test has the advantages of being quick and does not require special equipment or training, it will be easier to perform as part of routine health management. In addition, a review study shows a significant correlation between TUG test results and a history of falls (Beauchet et al., 2011). On the other hand, in predicting PD risk, a cohort study involving 1,497,093 older adults over 3.5 years found that participants with slower TUG test results—defined as 20 s for abnormal outcomes—had a significantly increased risk of developing PD compared to those with normal TUG test results (Yoo et al., 2020).

Exercise has been shown to improve TUG in PD (Gobbi et al., 2009). However, the effect of different forms of exercise on improving TUG in PD patients remains controversial. For example, a recent meta-analysis result by Zhen et al. (2022) found aerobic exercise can improve TUG in PD patients. But, Sousa et al. (2017) showed that mixed exercise can improve TUG in PD patients more effectively than aerobic exercise. Not only that, in terms of anaerobic resistance training the systematic review results of Lima et al. confirmed resistance exercise as a more effective modality to improve TUG in PD (Lima et al., 2013). Meanwhile, it is worth exploring Meng et al.’s study which found that both resistance exercise and mind–body exercise can improve identically TUG in PD (Ni et al., 2016). Interestingly, the RCTs of Palamara et al. (2017) found that aquatic exercise was more effective than land exercise in improving TUG in PD. In addition, sensory training has been gaining popularity in recent years, in a network meta-analysis, the study results found that sensory training had a superior effect on improving TUG in PD patients (Qian et al., 2023).

The reason for the different effects of different exercise types to improve TUG may be due to differences in exercise dosage. And, the amount of exercise dosage is strongly associated with sustained improvement in health (Foulds et al., 2014). In addition, the result of Gallardo et al. showed a nonlinear dose–response relationship between exercise and cognitive improvement in old adults, and their studies, it is also the first time determining the exercise dose by task metabolic equivalents (Gallardo-Gómez et al., 2022, 2023). At the same time, a recent meta-analysis study results found that exercise interventions recommended by the American College of Sports Medicine (ACSM), which include elements of flexibility, cardiovascular endurance, muscle strength, functional training, and motor control, can significantly enhance motor function, activity ability in PD when adhered to with high compliance (Cui et al., 2023). However, the optimal exercise form and exercise dose to improve TUG in PD patients has not been clearly defined in previous studies. In our study, we continue to put the exercise dose as calculated as task metabolic equivalents for an intervention (Willis et al., 2024). To determine the relationship between exercise dose and TUG improvement.

We utilize advanced techniques in this systematic review and network meta-analysis, including model-based dose–response network meta-analysis within a Bayesian framework (Pedder, 2021) to investigate the relationship between various exercise interventions and TUG in PD patients. We acknowledge that statistically significant improvements in TUG may not always reflect clinically meaningful changes. Hence, our analysis emphasizes assessing clinical relevance alongside statistical significance. Additionally, we aim to determine the minimum clinically important difference (MCID) for TUG and identify optimal exercise dosages for clinically meaningful improvements. Our research will contribute significantly to evidence-based exercise guidelines for managing motor symptoms in PD, aiding healthcare professionals in decision-making.

## Method

This systematic review and network meta-analysis (NMA) were registered with the International Prospective Register of Systematic Reviews (PROSPERO) (CRD42024506968) and This NMA was reported by the preferred Reporting Items for Systematic Review and Meta-analysis Protocols statement extension for PRISMA-NMA checklist (Page et al., 2021).

### Search strategy

Literature was systematically retrieved through comprehensive searches conducted in PubMed, Medline, Embase, PsycINFO, Cochrane Central Register of Controlled Trials, and Web of Science, from the inception of each database to February 5th, 2024. The following subject heading and keyword were used for electronic searching: (“Parkinson Disease” or “Parkinson” or “Parkinson’s Disease”) AND (“exercise” or “Exercises” or “Physical Activity” or “Training” or “endurance training” or “Tai Chi” or “yoga” or “Balance” or “Resistance” or “Walking” or “Dance” or “Aerobic”) AND (“TUG” or “Timed up and Go”) AND (“Randomized controlled trial” or “controlled clinical trial” or “randomized”). Detailed search strategies for each database and platform can be found in Supplementary File 1.

In addition, to ensure no relevant studies were overlooked, we meticulously reviewed the reference lists of all selected articles and the bibliographies of systematic reviews published within the past 5 years. The screening process for titles/abstracts and full texts were rigorously carried out by two independent investigators (JYW and LZ), with any discrepancies resolved through discussion or, if necessary, by consulting a third author (YY) for adjudication.

### Eligibility criteria and study selection

Studies were selected based on specific inclusion criteria: (1) Participants had to be diagnosed with Parkinson’s Disease (PD), with a mean age of 50 years or older, and at Hoehn and Yahr stages below 4; (2) The intervention involved any type of exercise, encompassing 9 distinct exercise modalities as detailed in Supplementary File 2; (3) Comparators included those receiving no intervention, standard care, educational sessions on the disease, or active control, which could involve a different exercise type from the intervention group or the same exercise type but at a varied dosage; (4) Outcomes had to include Timed up and go test (TUG) (Podsiadlo and Richardson, 1991); (5) The study design must be a randomized controlled trial (RCT). Exclusion criteria were applied to studies that: (1) focused solely on the acute effects of exercise; (2) incorporated mixed interventions from different disciplines (e.g., combining exercise with repetitive transcranial magnetic stimulation); (3) lacked clear descriptions of exercise types or sufficient details to calculate exercise dosage; (4) did not provide mean values and standard deviations in their results, or failed to respond to our data requests. Following these criteria, two independent reviewers (JYW and LZ) meticulously screened the titles, abstracts, and full texts of potentially relevant studies to determine their suitability for inclusion.

### Data extraction

Two reviewers (JYW and LZ) meticulously extracted critical information from each publication, including authorship, title, publication year, and journal name, along with specific data on the study population such as the number of participants, and their demographic details (age and sex). The nature of the interventions and the outcome measures employed were also cataloged (detailed in Supplementary File 3). To calculate effect sizes, data on the change scores (difference between endpoint and baseline scores), standard deviations, and the number of participants in each group were collected. In instances where the mean changes and standard deviations were not directly reported, they were estimated by the guidelines provided in the Cochrane Handbook (Higgins et al., 2019). To meet the data analysis requirements of the Dose–Response Network meta-analysis package in the R program, we also converted the standard errors [SE = SD /SQRT (Sample size)] (Watt et al., 2022). Should the required data not be obtainable through these methods, we committed to contacting the original authors up to four times over 6 weeks to request the necessary information.

### Data setting and management

First, we assigned specific codes to interventions based on the type of exercise performed. These codes included: “Aerobic exercise (AE),” “Aquatic exercise (AQE),” “Balance and gait training (BGT),” “Resistance Training (RT),” “Dance,” “Mixed exercise (Mix, combination of 2 or more special exercise types),” “Sensory Exercise (SE),” Treadmill Training (TT), “Mind Body Exercise (MBE: including “Qigong,” “Tai Chi,” and “Yoga”). Next, interventions were further categorized based on their specific type and dose combinations, expressed in terms of METs-min/week (Metabolic Equivalent of Task (METs)). Not only, by calculating METs-min consumed per week, our study took into account not only the duration and frequency of exercise *(METs-min/week = duration minute × times - pre-week × MET value)* but also the intensity of exercise, which is critical to assess its impact on health outcomes (Ainsworth et al., 2011; Ferguson, 2014; Wasfy and Baggish, 2016). To facilitate network analysis and ensure connectivity, we applied approximate values of 250, 500, 750, 1,000, or 1,200 MET-min/week for exercise dosages, as previously employed in similar studies (Gallardo-Gómez et al., 2022). This step was essential for conducting the network meta-analysis as outlined by J.P.T. Higgins et al. (2012). We provided a detailed inclusion of study-specific data information about different exercise METs and approximate values in Supplementary Table 2.

### Data synthesis

In our analysis, we utilized the R statistical environment (Version 4.3.0)^1^ and employed the ‘MBNMAdose’ package (Pedder, 2021) for conducting Model-Based Network Meta-Analysis (MBNMA). We specifically implemented a random-effects Bayesian MBNMA approach to synthesize data and assess the dose–response relationship between exercise dosage and TUG. Our assessment involved several key components, including an evaluation of network connectivity following the method described by Donegan et al. (2013), a model assessment using the approach outlined by Wheeler et al. (2010), and an examination of data consistency as per White et al. (2012) (details provided in Supplementary Files 4, 5). All effect sizes were reported as mean differences (MD), with 95% credible intervals (CrI) used to assess the credibility of our estimates. In the process of selecting an appropriate dose–response model, we compared fit indices, including the Deviance Information Criterion (DIC), between-study standard deviation, number of parameters in the model, and residual values, as suggested by Evans (2019). Ultimately, we opted for restricted cubic splines to evaluate the non-linear dose–response association, as detailed in Supplementary File 6.

To further enhance the clinical relevance of our findings, we conducted an estimation of the exercise dosage or range of dosages required to achieve the Minimum Clinically Important Difference (MCID), as recommended by Bernstein and Mauger (2016). In our analysis, we applied a distribution-based method to establish a consolidated MCID value for the TUG test, following the approach outlined by Watt et al. (2021). Our results indicated that the MCID for the TUG test could be estimated as a reduction of −2.7 s when considering a 0.4 standard deviation (SD) threshold, or a reduction of −3.4 s at a 0.5 SD level. However, to provide clinicians with more robust and clinically meaningful guidance, we ultimately selected an MCID of −3.4 s at the 0.5 SD threshold. This choice reflects our commitment to delivering rigorous and valuable recommendations for clinical practice, ensuring that our findings can be effectively applied to benefit patients.

### Risk of bias and quality of evidence

Two reviewers (HYZ and XYF), meticulously evaluated and rated the included studies by the Cochrane Risk of Bias 2.0 criteria, the study-assessment items included randomized sequence generation, bias due to deviation from the intended intervention, incomplete data, measurement bias, selective bias in reporting results, which as detailed by Sterne et al. (2019). In instances where discrepancies arose, they were resolved through thorough discussion or by seeking the input of a third reviewer, YY, to reach a consensus.

## Results

### Characteristics of included studies

**Figure 1:**
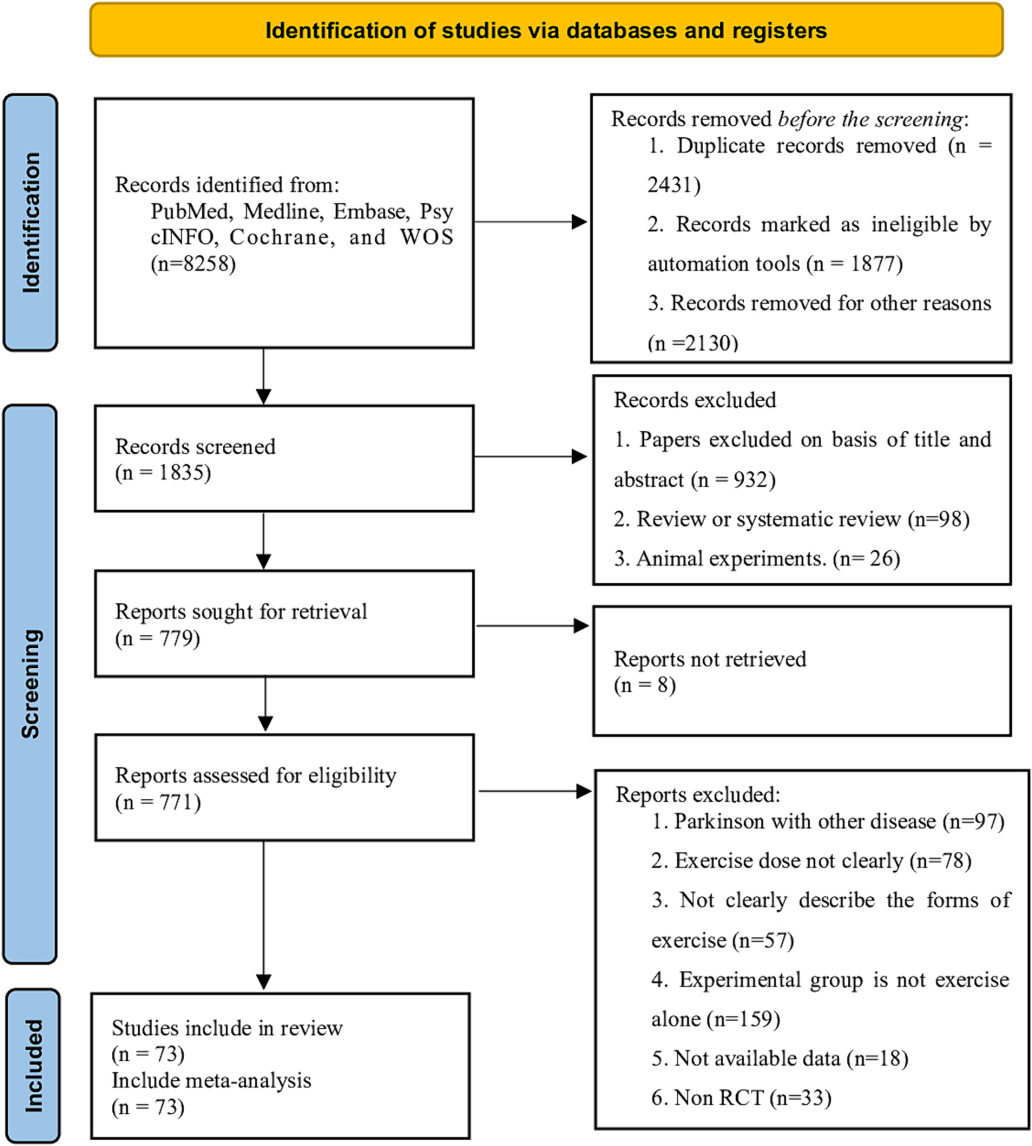
PRISMA flow diagram of the search process for studies. *RCT* randomized controlled trials.

A total of 5,258 articles were retrieved from the databases and through hand-searching. After removing duplicates, we screened the citations by title and abstract, considering 771 potentially eligible articles, and subsequently searched for their full texts. After excluding studies that did not meet the inclusion criteria, 73 studies were included in our analysis (Figure 1). These studies involved 3,354 participants (1,963 males), all of whom had PD and were aged between 51.6 to 72 years, with a mean disease duration of 6.34 years (SD = 2.34) and a mean Hoehn and Yahr stage of 3.25 (SD = 0.35). The exercise period ranged from 2 to 48 weeks (mean = 9.3 weeks, SD = 6.1), the frequency of exercise training per week ranged from 1 to 7 sessions (mean = 2.89 sessions, SD = 1.22), and the duration of each session ranged from 15 to 90 min (mean = 49.0 min, SD = 12.6). The characteristics of all included populations are detailed in Supplementary Table 1.

### Network connectivity

**Figure 2:**
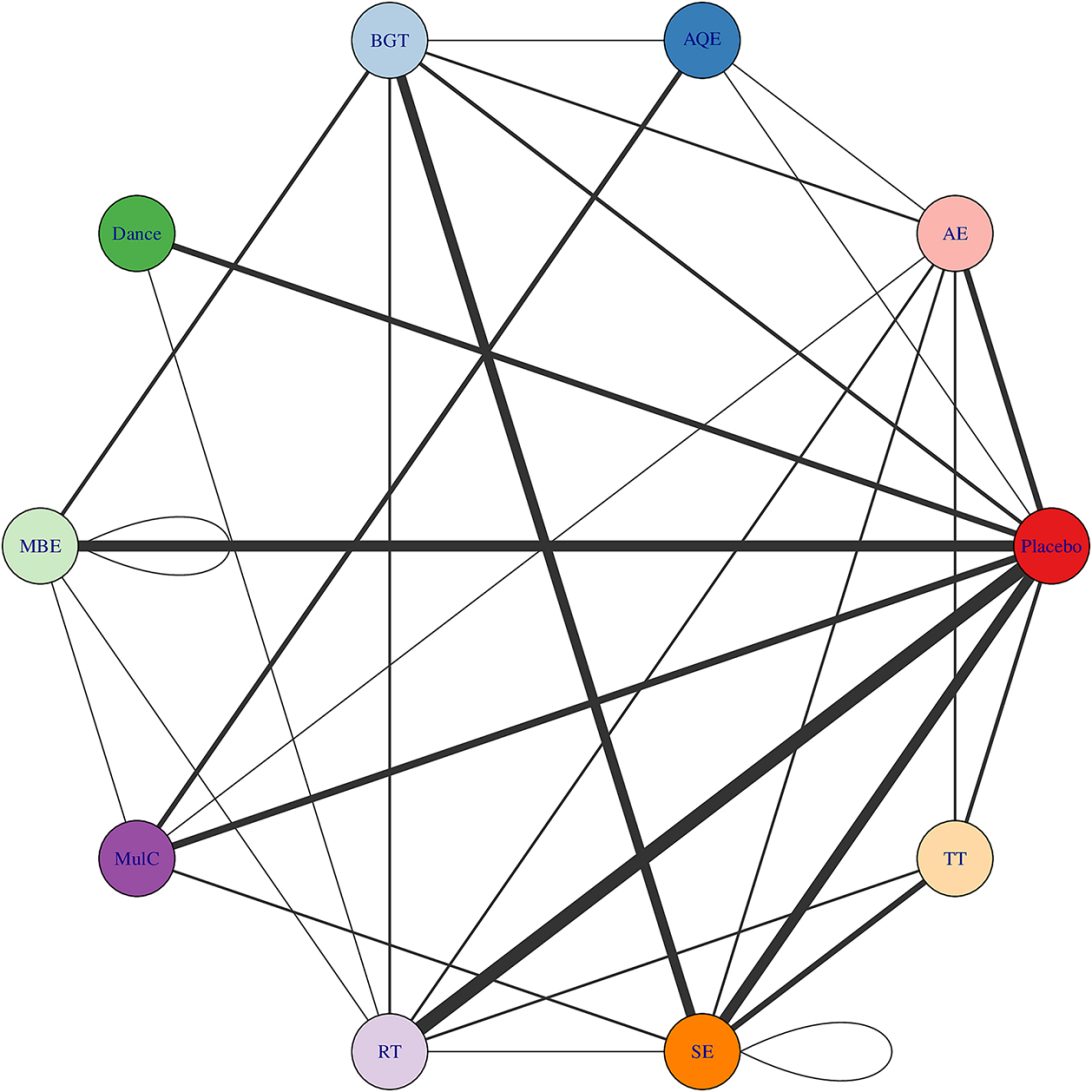
Treatment-level and agent-level network plot. The first value indicates the specific intervention and the second one is the corresponding dose of that intervention. AE Aerobic Exercise, AQE Aquatic Exercise, BGT Balance and Gait Training, Dance, MBE Mind–body Exercise, CON Control group, MulC Multicomponent Exercise Program, RT Resistance Training, TT Treadmill Training, SE Sensory Exercise.

**Figure 3:**
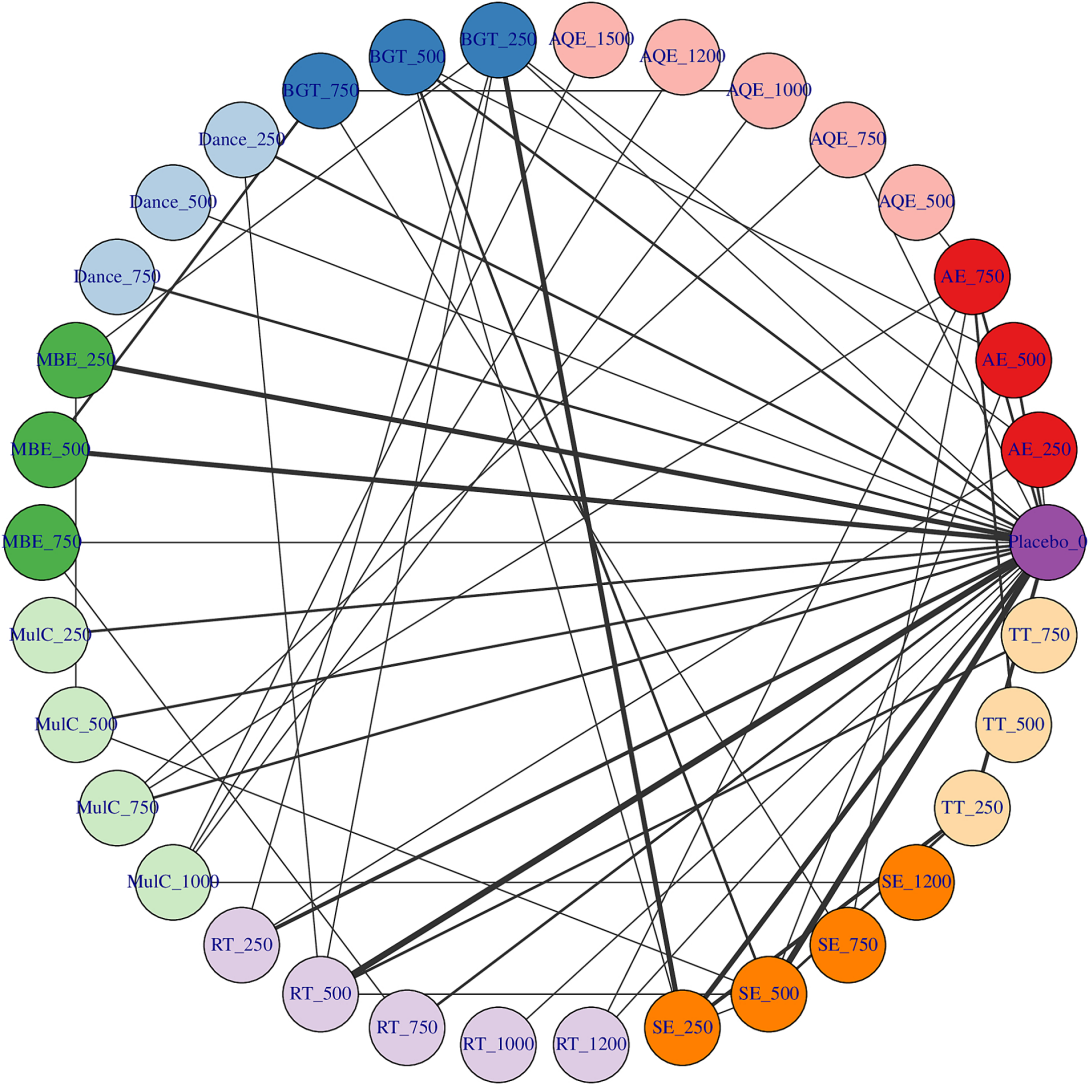
Dose–response association between agent-level dose and change in TUG in Parkinson’s disease patients, the exercise dose distribution is represented by the green part in our study.

Whether or not connectivity is met determines the basis of NMA. When direct comparison is not possible, lack of connectivity can lead to low statistical power and misleading results (Rouse et al., 2017). The results showed that there was no connectivity deficit in the two networks, thus ensuring the accuracy of the analysis (Figures 2, 3).

### Dose–response relationships

Figure 3 shows the dose–response relationship of different exercise types improving TUG performance. We described in detail the different exercise types and doses to improve the TUG performance in patients with PD. In 7 studies involving 99 patients with PD in AQE, the graphical results showed a nonlinear improvement in the mean value of TUG performance changes in PD patients with increasing doses of AQE. The effective dose of AQE ranges from 790 to 1,500 METs-min/week for improving TUG performance, and the optimal AQE dose is estimated at 1500 METs-min/week (MD: −8.359, 95Crl: −1.398 to −2.648). Significantly, we identified the MCID among the exercise doses for AQE, in which AQE exceeding 970 METs-min/week was found to be a significant clinical effect for improving the TUG performance.

At the same time, in 12 studies involving 237 patients with PD in Mul_C, Where the effective range of Mul_C was estimated at 570 METs-min/week to 1,000 METs-min/week, and the optimal Mul_C dose was estimated at 1000 METs-min/week (MD: −4.551, 95%Crl: −8.083 to −0.946), For MCID, when the Mul_C dose exceeding 870 METs-min/week was found to be a significant clinical effect for improving the TUG performance. On the other hand, in 25 studies involving 570 PD patients with PD in SE, the effective range of SE was estimated at 350 ~ 1,200 METs-min/week, and the optimal SE dose was estimated at 1200 METs-min/week (MD: −5.145, 95Crl: −9.643 to −0.472), For MCID, when the SE dose exceeding 910 METs-min/week was found to be a significant clinical effect.

In 18 studies involving 396 patients with PD in RT, we found that RT dose–response results showed an inverted U shape and had optimal doses for improving the TUG performance. In this result, the effective dose of RT ranges from 85 to 980 METs-min/week for improving TUG performance, and the optimal RT dose was estimated at 610 METs-min/week (MD: −2.187, 95Crl: −3.161 to −1.278) at the same time, we also did not find significant clinical effects in the RT dose.

**Figure 4:**
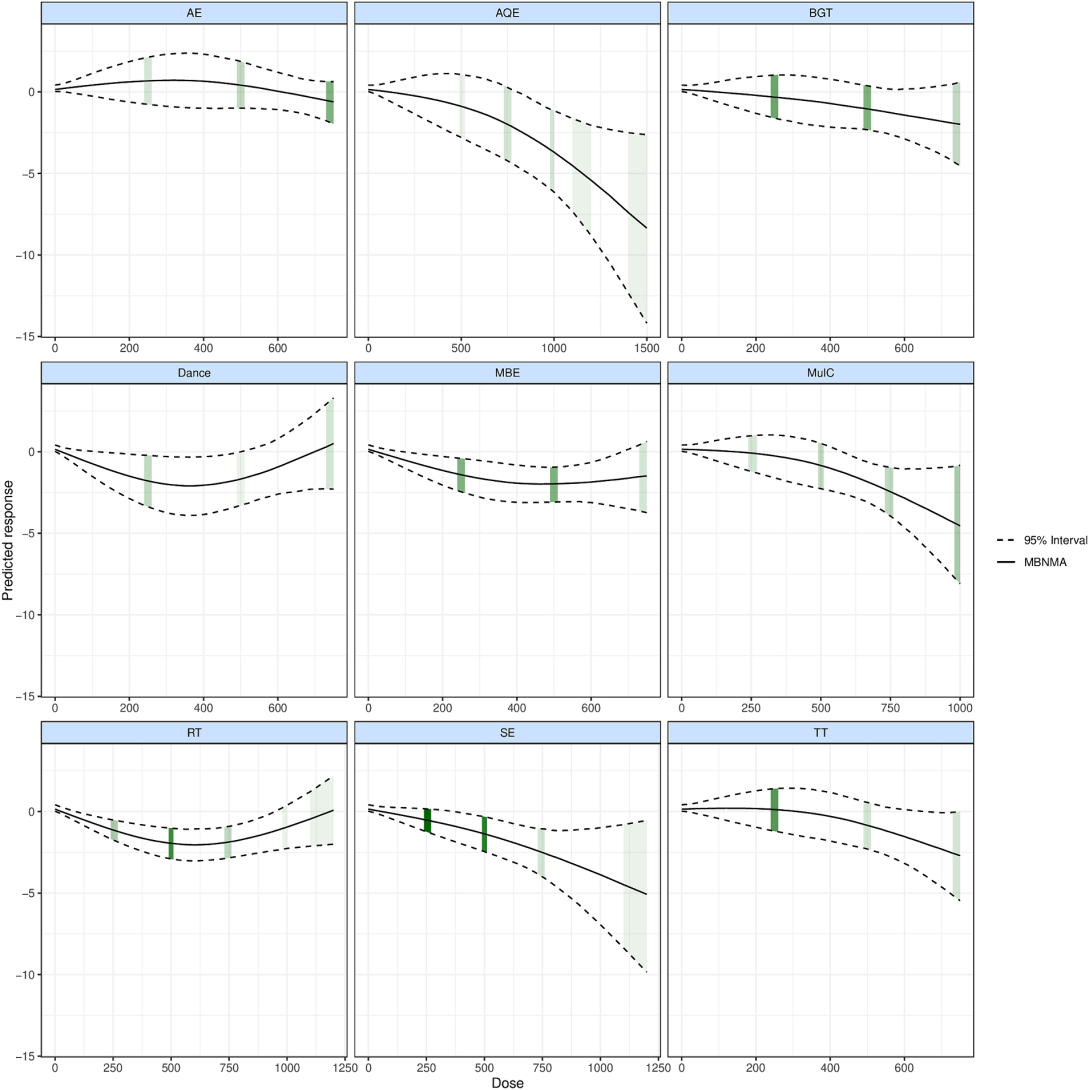
Dose–response association between treatment-level dose and change in motor symptoms in Parkinson’s disease patients.

In 11 studies involving 169 patients with PD participated in AE, 16 studies involving 236 patients with PD participated in BGT, 6 studies involving 95 patients with PD participated in Dance, 14 studies involving 348 patients with PD participated in MBE, and 12 studies involving 227 patients with PD participated in TT. These studies were worth attention that none of the exercise doses for AE, BGT, Dance, MBE, and TT exceeded 750 METs-min/week (Figure 3 Shades of green represent the sample size for distributing exercise doses, with larger sample sizes being darker and vice versa). At the same time, we also found that AE, BGT, Dance, and TT did not effectively improve the TUG performance at any dose. However, it is interesting to note that MBE the effective dose of MBE ranges from 130 to 750 METs-min/week for improving TUG performance and 750 METs-min/week (MD: −2.822, 95%Crl: −4.604 to −0.996) appears to be the optimal MBE dose for improving the TUG performance in PD patients. In addition, For MCID, it is worth exploring a point in which we did not find significant clinical effects in the MBE dose (Figure 4).

### Risk of bias and quality of evidence

**Figure 5:**
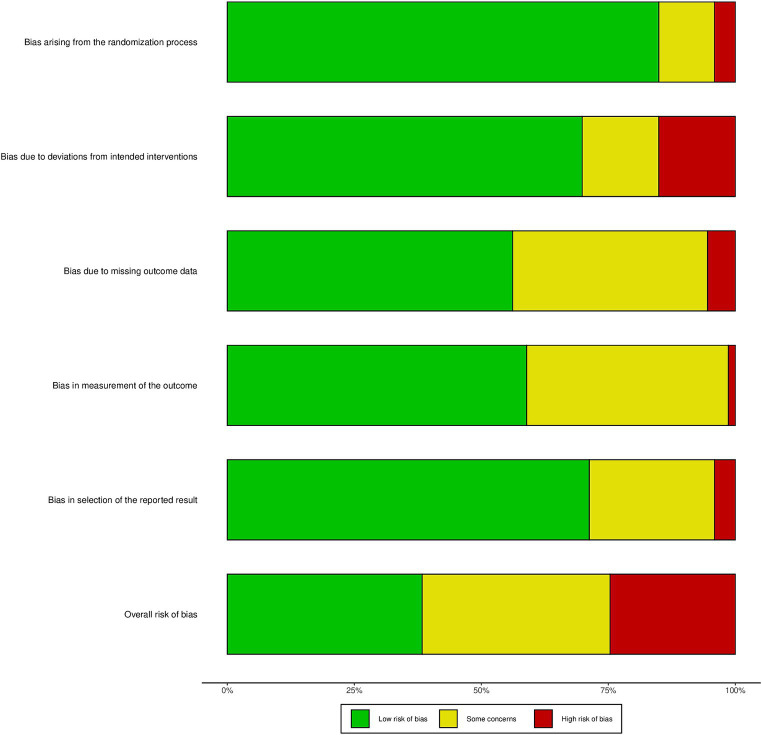
Cochrane risk of tool.

Overall, 22 studies (40%) were classified a low risk of bias,20 studies (38%) were classified unclear risk of bias, and 12 studies (23%) were classified high risk of bias, Figure 5 shows the result of Cochrane Risk of Bias Tool and Study-level risk of bias assessments are presented in Supplementary File 7. According to the GRADE system, the overall quality of the evidence was moderate.

## Discussion

### Main finding

In this dose–response study, which incorporated 73 studies involving 3,354 patients with PD, we uncovered a nonlinear dose–response relationship between various exercise modalities and improvements in TUG performance. Among the nine types of exercises evaluated, AQE, Mul_C, SE, RT, and MBE demonstrated significant improvements in TUG function in PD patients. Notably, AQE emerged as the most effective modality, with an optimal dosage identified at 1500 METs-min/week. Further analysis into the dosage range necessary to achieve the Minimum Clinically Important Difference (MCID) in PD patients revealed that AQE, Mul_C, and SE all significantly enhanced TUG performance, with respective MCID dosages of 970, 870, and 910 METs-min/week. Additionally, RT exhibited an inverted U-shaped dose–response curve, indicating an effective dosage range from 85 to 980 METs-min/week for improving TUG performance. Moreover, MBE was effective within a dosage range of 130 to 750 METs-min/week, suggesting its potential to significantly enhance TUG performance at lower dosages in PD patients.

### Strengths

This study reinforces that AQE is the optimal exercise modality for improving TUG performance in PD patients, with an established effective dosage of 1,500 METs-min per week. Consistent with prior research (Volpe et al., 2014; Qian et al., 2023), our findings highlight AQE’s significant benefits in enhancing posture stability and motor symptoms, thus improving balance capabilities in PD patients. The TUG test, a standard tool for assessing functional balance in daily activities, reflects balance and posture control. We propose that the therapeutic benefits of AQE, derived from the buoyancy, resistance, and warmth of water, create an advantageous environment for PD patients. Water buoyancy alleviates the effect of gravity, easing the load on weight-bearing joints, reducing pain, and enhancing mobility (Al-Qubaeissy et al., 2013), while also diminishing fall anxiety (Gomes Neto et al., 2020), which encourages safer, larger movements. The resistance of water increases the effort required for movement, engaging multiple muscle groups crucial for motor function and stability. Additionally, the warmth of water relaxes muscles, reduces spasticity, and promotes circulation, aiding flexibility and mobility, particularly beneficial for those with muscle rigidity and bradykinesia (Malanga et al., 2015; An et al., 2019). Consequently, AQE’s multifaceted therapeutic effects render it superior to other exercise forms in enhancing TUG performance.

Despite AQE’s effectiveness, its implementation faces challenges such as site restrictions and high costs, limiting accessibility for PD patients. This study explored the impacts of Mul_C and SE as viable alternatives, achieving the MCID at dosages of 870 and 910 METs-min/week, respectively. Mul_C integrates diverse exercise elements—such as aerobic and strength training, balance, and flexibility exercises—and promotes neuroplasticity, enhancing both motor and non-motor symptoms (Zhen et al., 2022). SE, through targeted sensory inputs like visual, auditory, and proprioceptive cues, significantly improves motor control and postural stability, optimizing movement strategies and reducing fall risk (Abbruzzese et al., 2016; Carpinella et al., 2017; Beck et al., 2020). Given the challenges associated with AQE, Mul_C, and SE present effective, lower-cost alternatives that improve motor function and quality of life in PD patients. Future research should investigate these modalities across diverse PD populations to develop optimal, comprehensive rehabilitation strategies.

This study found that increased doses of AQE, Mul_C, and SE led to nonlinear improvements in TUG test scores for PD patients, suggesting that appropriate exercise dosage can enhance motor functions. However, an optimal balance in exercise dosage is essential to avoid overtraining risks, such as increased fatigue or adverse reactions, which can impair rehabilitation (Smith, 2000; da Rocha et al., 2019). The limited data available restricted our ability to pinpoint the optimal exercise dosage for balancing efficacy and safety, underscoring the need for further research into various exercise dosages and their impacts on motor function in PD patients.

The study also revealed a “U-shaped” relationship between RT dosage and TUG performance improvements, with 610 METs-min/week identified as optimal. Research supports that RT enhances muscle strength and motor coordination, which are crucial for PD patients at risk of gait instability and falls due to muscle rigidity and bradykinesia (Brienesse and Emerson, 2013; Chung et al., 2016; Li et al., 2020). However, excessive RT can cause muscle over-fatigue and damage, particularly in PD patients with compromised recovery capabilities due to neurodegenerative changes, highlighting the importance of optimizing RT dosage for safety and effectiveness (Siciliano et al., 2018).

Furthermore, our findings indicate that MBE, even at a low dosage of 130 MET-min/week, significantly benefits PD patients, making it a viable option for those less active or non-adherent to exercise regimes. MBE practices like Tai Chi, Qigong, and Yoga focus on balance and coordination, integrating mind and body through movement and deep breathing, and potentially improving neuroplasticity and alleviating symptoms (Benke et al., 1998; Tessitore et al., 2002; Shen et al., 2016; Kwok et al., 2019). This suggests that MBE’s accessible approach could significantly enhance the quality of life for PD patients, warranting further promotion and research.

### Limitations

While this NMA incorporated high-quality randomized controlled trials, several limitations warrant consideration. Firstly, the study is subject to the inherent limitations of the included studies in the meta-analysis, such as variability within the PD patient cohorts, the types and dosages of exercise interventions employed, and the outcomes assessed. Additionally, this study only included published randomized controlled trials, which may introduce publication bias, as studies yielding significant results are more likely to be published. Moreover, the methodology used to determine the optimal exercise dosage relied on approximations (e.g., 250, 500, 750, 1,000, or 1500MET-min/week), which might oversimplify the situation and affect the accuracy of the results. Lastly, the study did not evaluate the impact of individual patient characteristics (such as age, ethnic background, etc.) on the efficacy of exercise interventions, nor did it conduct subgroup analyses, which could limit the applicability of the study findings to individual patients. Future research should build on the results of this study to determine personalized exercise programs for PD patients with different individual characteristics, aiming to maximize the improvement of TUG function and enhance their quality of life.

### Clinical implications and directions for future research

The clinical significance of our study was to systematically evaluate the impact of different exercise interventions on TUG test performance in PD patients, use task metabolic equivalents as the benchmark for exercise intensity assessment, determine the optimal exercise form and dosage, and provide evidence-based exercise prescriptions for clinical practice.

Our study outlines new directions for future research. We propose that long-term studies be designed and implemented to evaluate the sustained effects of various types and doses of exercise interventions on TUG performance in patients with PD. Such studies will help ascertain the long-term benefits of these interventions on daily functioning and quality of life. Additionally, personalized exercise programs should be developed and validated for PD patients at different stages of the disease, of varying ages, and with diverse physical conditions. Customized exercise programs can more effectively meet individual patient needs and maximize the benefits of exercise. Furthermore, future studies should include subgroup analyses to explore how factors such as gender, race, and disease severity affect the efficacy of exercise interventions. This will enhance the precision of exercise recommendations and ensure that all PD patients can derive benefits from exercise. In terms of exercise dose, it is crucial to employ more sophisticated dose–response models to accurately determine the relationship between exercise dose and TUG performance. This approach will provide more precise and practical exercise dosing recommendations for clinical practice. It is also important to rigorously assess the potential adverse effects of exercise interventions to ensure the safety of exercise regimens. Detailed recording and analysis of adverse events are essential to provide scientifically sound and safe exercise prescriptions for PD patients.

## Conclusion

This study has identified specific exercise modalities and dosages that significantly improve TUG performance in PD patients. AQE emerged as the most effective modality with an optimal dosage of 1,500 METs-min/week, while MBE demonstrated significant benefits at lower dosages, offering a practical option for patients with varying exercise capacities. The study also highlighted a nuanced “U-shaped” dose–response relationship for RT, pinpointing an optimal range that balances efficacy with the risk of overtraining. These findings advocate for the integration of tailored exercise programs into PD management strategies, emphasizing the need for personalized prescriptions to maximize patient outcomes.

## Data availability statement

The original contributions presented in the study are included in the article/Supplementary material, further inquiries can be directed to the corresponding authors.

## Author contributions

YYu: Writing – original draft, Writing – review & editing, Validation. JW: Writing – review & editing. GW: Conceptualization, Investigation, Writing – review & editing. TW: Methodology, Supervision, Writing – review & editing. HZ: Funding acquisition, Visualization, Writing – review & editing. XF: Software, Supervision, Writing – review & editing. LW: Methodology, Project administration, Writing – review & editing. XC: Data curation, Writing – review & editing. RX: Visualization, Writing – review & editing. LZ: Investigation, Software, Writing – review & editing. S-CL: Methodology, Writing – review & editing. YYa: Formal analysis, Investigation, Project administration, Software, Supervision, Writing – review & editing.

## Footnotes

^1^ www.r-project.org
