# Supplementary material for: Optimal dosage ranges of various exercise types for enhancing timed up and go performance in Parkinson’s disease patients: a systematic review and Bayesian network meta-analysis
Source: Front Aging Neurosci. 2024 Jun 24;16:1399175. doi: 10.3389/fnagi.2024.1399175 (PMC11234838; doi:10.3389/fnagi.2024.1399175)
Supplement: Supplementary file 1 [file Data_Sheet_1.docx]

**Optimal Dosage Ranges of Various Exercise Types for Enhancing Timed Up and Go Performance in Parkinson's Disease Patients: A Systematic Review and Bayesian Network Meta-analysis**

[Supplementary file 1: Search Strategy 2](#_Toc158037839)

[1.1 Database: PubMed <inception to February 5^th^ 2024> 2](#_Toc158037840)

[1.2 Database: Ovid MEDLINE(R) <1946 to February 5^th^ 2024> 6](#_Toc158037841)

[1.3 Database: Embase <1974 to 8](#_Toc158037842)

[1.4 Database: PsycINFO <1806 to February 5^th^ 2024> 10](#_Toc158037843)

[1.5 Cochrane 12](#_Toc158037844)

[1.6 Database: Web of Science <1965 to February 5^th^ 2024> 13](#_Toc158037845)

[Supplementary File 2: Definitions of exercise types and non-exercise training control 16](#_Toc158037846)

[Supplementary File 3: Characteristics of included studies 18](#_Toc158037847)

[**List of included studies** 28](#_Toc158037848)

[Supplementary File 4: Assessment of Model Consistency 36](#_Toc158037849)

[**Table 4.1. Consistent and UME models fit comparison** 36](#_Toc158037850)

[Supplementary File 5: Node-splitting analysis of inconsistency 37](#_Toc158037851)

[**Table 5.1 Node-splitting analysis of inconsistency** 37](#_Toc158037852)

[Supplementary File 6: Non-linear functions and models fit comparison 41](#_Toc158037853)

[**Table 6.1** Models fit Comparison 42](#_Toc158037854)

[Supplementary File 7: Study-level risk of bias analysis 44](#_Toc158037855)

# **Supplementary file 1: Search Strategy**

## 1.1 Database: PubMed <inception to February 5^th^ 2024>

***Search Strategy:***

| #25 | Search: ((Parkinson disease[MeSH Terms]) AND ((((((((((exercise*[MeSH Terms]) OR (resistance training[MeSH Terms])) OR (Tai Ji[MeSH Terms])) OR (Qigong[MeSH Terms])) OR (Exercise Movement Techniques[MeSH Terms])) OR (Yoga[MeSH Terms])) OR (Virtual Reality[MeSH Terms])) OR (hydrotherapy[MeSH Terms])) OR (Dance Therapy[MeSH Terms])) OR ("aerobic exercise" or "aquatic exercise" or "balance training" or "body weight support treadmill" or "gait **training" or "high-speed resistance training" or "multicomponent exercise program" or "multidisciplinary exercise program" or "Nordic Walking" or Physiotherapy or pilates or "power training" or "Robotic-assisted gait training" or stretch or Tango or "treadmill training" or "walking" or "whole body vibration"))) AND ((((((((randomized controlled trial[**Publication Type]) OR (controlled clinical trial[Publication Type])) OR (randomized[Title/Abstract])) OR (placebo[Title/Abstract])) OR (randomly[Title/Abstract])) OR (trial[Title])) OR (clinical trials as topic[MeSH Terms])) NOT ((animals[MeSH Terms]) NOT (humans[MeSH Terms]))) | 890 |
| --- | --- | --- |
| #24 | Search: (((((((randomized controlled trial[Publication Type]) OR (controlled clinical trial[Publication Type])) OR (randomized[Title/Abstract])) OR (placebo[Title/Abstract])) OR (randomly[Title/Abstract])) OR (trial[Title])) OR (clinical trials as topic[MeSH Terms])) NOT ((animals[MeSH Terms]) NOT (humans[MeSH Terms])) | 1,341,164 |
| #23 | Search: ((((((randomized controlled trial[Publication Type]) OR (controlled clinical trial[Publication Type])) OR (randomized[Title/Abstract])) OR (placebo[Title/Abstract])) OR (randomly[Title/Abstract])) OR (trial[Title])) OR (clinical trials as topic[MeSH Terms]) | 1,449,600 |
| #22 | Search: (((((((((exercise*[MeSH Terms]) OR (resistance training[MeSH Terms])) OR (Tai Ji[MeSH Terms])) OR (Qigong[MeSH Terms])) OR (Exercise Movement Techniques[MeSH Terms])) OR (Yoga[MeSH Terms])) OR (Virtual Reality[MeSH Terms])) OR (hydrotherapy[MeSH Terms])) OR (Dance Therapy[MeSH Terms])) OR ("aerobic exercise" or "aquatic exercise" or "balance training" or "body weight support treadmill" or "gait training" or "high-speed resistance training" or "multicomponent exercise program" or "multidisciplinary exercise program" or "Nordic Walking" or Physiotherapy or pilates or "power training" or "Robotic-assisted gait training" or stretch or Tango or "treadmill training" or "walking" or "whole body vibration") | 551,542 |
| #21 | Search: (animals[MeSH Terms]) NOT (humans[MeSH Terms]) | 4,815,925 |
| #20 | Search: humans[MeSH Terms] | 19,183,084 |
| #19 | Search: animals[MeSH Terms] | 23,999,009 |
| #18 | Search: clinical trials as topic[MeSH Terms] | 355,600 |
| #17 | Search: trial[Title] | 238,308 |
| #16 | Search: randomly[Title/Abstract] | 356,459 |
| #15 | Search: placebo[Title/Abstract] | 223,336 |
| #14 | Search: randomized[Title/Abstract] | 561,707 |
| #13 | Search: controlled clinical trial[Publication Type] | 617,986 |
| #12 | Search: randomized controlled trial[Publication Type] | 528,725 |
| #11 | Search: "aerobic exercise" or "aquatic exercise" or "balance training" or "body weight support treadmill" or "gait training" or "high-speed resistance training" or "multicomponent exercise program" or "multidisciplinary exercise program" or "Nordic Walking" or Physiotherapy or pilates or "power training" or "Robotic-assisted gait training" or stretch or Tango or "treadmill training" or "walking" or "whole body vibration" | 361,398 |
| #10 | Search: Dance Therapy[MeSH Terms] | 396 |
| #9 | Search: hydrotherapy[MeSH Terms] | 20,257 |
| #8 | Search: Virtual Reality[MeSH Terms] | 2,684 |
| #7 | Search: Yoga[MeSH Terms] | 3,002 |
| #6 | Search: Exercise Movement Techniques[MeSH Terms] | 8,700 |
| #5 | Search: Qigong[MeSH Terms] | 229 |
| #4 | Search: Tai Ji[MeSH Terms] | 1,183 |
| #3 | Search: resistance training[MeSH Terms] | 9,538 |
| #2 | Search: exercise*[MeSH Terms] | 297,336 |
| #1 | Search: Parkinson disease[MeSH Terms] | 69,308 |

## 1.2 Database: Ovid MEDLINE(R) <1946 to February 5^th^ 2024>

***Search Strategy: --------------------------------------------------------------------------------***

1 Parkinson$.mp. (136173)

2 exp Parkinson disease/ (69312)

3 (aerobic exercise or aquatic exercise or balance training or body weight support treadmill or Dance Therapy or exercise$ or Exercise Movement Techniques or gait training or high-speed resistance training or hydrotherapy or multicomponent exercise program or multidisciplinary exercise program or Nordic Walking physiotherapy pilates or power training or Qigong or resistance training or Robotic-assisted gait training or stretch or tai ji or Tango or treadmill training or walking or Virtual Reality or whole body vibration or Yoga).mp. (540012)

4 exp resistance training/ (9532)

5 exp exercise$/ (206975)

6 exp tai ji/ (1182)

7 exp Qigong/ (228)

8 exp Exercise Movement Techniques/ (8695)

9 exp Yoga/ (2999)

10 exp Virtual Reality/ (2682)

11 exp hydrotherapy/ (20254)

12 exp Dance Therapy/ (396)

13 randomized controlled trial.pt. (527440)

14 controlled clinical trial.pt. (94123)

15 randomized.ab. (517037)

16 clinical trials as topic.sh. (195553)

17 randomly.ab. (355668)

18 trial.ti. (238446)

19 exp clinical trial/ (888782)

20 exp randomized controlled trials/ (145969)

21 exp cross-over studies/ (49955)

22 (clinic$ adj2 trial).mp. (746815)

23 (random$ adj5 control$ adj5 trial$).mp. (770827)

24 (crossover or cross-over).mp. (100433)

25 randomi$.mp. (943880)

26 (random$ adj5 (assign$ or allocat$ or assort$ or reciev$)).mp. (256419)

27 1 or 2 (136173)

28 3 or 4 or 5 or 6 or 7 or 8 or 9 or 10 or 11 or 12 (586531)

29 13 or 14 or 15 or 16 or 17 or 18 or 19 or 20 or 21 or 22 or 23 or 24 or 25 or 26 (1770322)

30 27 and 28 and 29 (1246)

1.3 Database: Embase <1974 to February 5^th^ 2024>
***Search Strategy:***

--------------------------------------------------------------------------------

1 Parkinson$.mp. (219918)

2 exp parkinson disease/ (163492)

3 (aerobic exercise or aquatic exercise or balance training or body weight support treadmill or Dance Therapy or exercise$ or Exercise Movement Techniques or gait training or high-speed resistance training or hydrotherapy or multicomponent exercise program or multidisciplinary exercise program or Nordic Walking or Physiotherapy or pilates or power training or Qigong or resistance training or Robotic-assisted gait training or stretch or tai ji or Tango or treadmill training or walking or Virtual Reality or whole body vibration or Yoga).mp. (816955)

4 exp resistance training/ (20137)

5 exp exercise$/ (363106)

6 exp tai ji/ (3173)

7 exp Qigong/ (836)

8 exp Exercise Movement Technique/ (82933)

9 exp Yoga/ (8492)

10 exp Virtual Reality/ (18896)

11 exp hydrotherapy/ (3829)

12 exp Dance Therapy/ (527)

13 randomized.ab. (757440)

14 randomly.ab. (481077)

15 trial.ti. (332106)

16 exp clinical trial/ (1627821)

17 exp randomized controlled trials/ (200725)

18 exp cross-over studies/ (66963)

19 (clinic$ adj2 trial).mp. (1639410)

20 (random$ adj5 control$ adj5 trial$).mp. (961796)

21 (crossover or cross-over).mp. (123532)

22 randomi$.mp. (1323771)

23 (random$ adj5 (assign$ or allocat$ or assort$ or reciev$)).mp. (209949)

24 1 or 2 (219918)

25 3 or 4 or 5 or 6 or 7 or 8 or 9 or 10 or 11 or 12 (841979)

26 13 or 14 or 15 or 16 or 17 or 18 or 19 or 20 or 21 or 22 or 23 (2779253)

27 24 and 25 and 26 (2562)

## 1.4 Database: PsycINFO <1806 to February 5^th^ 2024>

***Search Strategy:***

| Set No. Searched for Databases Results | | | |
| --- | --- | --- | --- |
| S1 | Parkinson* | APA PsycInfo® | 39453 |
| S2 | mainsubject(parkinson disease) | APA PsycInfo® | 25842 |
| S3 | su((aerobic exercise or aquatic exercise or balance training or body weight support treadmill or Dance Therapy or exercise$ or Exercise Movement Techniques or gait training or high-speed resistance training or hydrotherapy or multicomponent exercise program or multidisciplinary exercise program or Nordic Walking or Physiotherapy or pilates or power training or Qigong or resistance training or Robotic-assisted gait training or stretch or tai ji or Tango or treadmill training or walking or Virtual Reality or whole body vibration or Yoga)) | APA PsycInfo® | 63328 |
| S4 | su(exercise$) | APA PsycInfo® | 39377 |
| S6 | su(physical activity) | APA PsycInfo® | 39490 |
| S7 | ab(randomized) | APA PsycInfo® | 83500 |
| S8 | ab(randomly) | APA PsycInfo® | 75844 |
| S9 | ti(trial) | APA PsycInfo® | 41193 |
| S10 | ab(clinical trial) | APA PsycInfo® | 51602 |
| S11 | ab(randomized controlled trials) | APA PsycInfo® | 36991 |
| S12 | ab(cross-over studies) | APA PsycInfo® | 2076 |
| S13 | ab(crossover studies) | APA PsycInfo® | 5363 |
| S14 | ab(randomi*) | APA PsycInfo® | 83917 |
| S15 | su(animals) | APA PsycInfo® | 459210 |
| S16 | S1 OR S2 | APA PsycInfo® These databases are searched for part of your query. | 39453 |
| S17 | S3 OR S4 OR "S5" | APA PsycInfo® These databases are searched for part of your query. | 63473 |
| S18 | S6 OR S7 OR "S8" OR "S9" OR "S10" OR "S11" OR "S12" OR "S13" OR "S14" | APA PsycInfo® These databases are searched for part of your query. | 120428 |
| S19 | S16 AND S17 | APA PsycInfo® These databases are searched for part of your query. | 1074 |
| S20 | S18 AND S19 | APA PsycInfo® These databases are searched for part of your query. | 277 |
| S21 | S20 NOT S15 | APA PsycInfo® These databases are searched for part of your query. | 251 |

## 1.5 Cochrane

#1  MeSH descriptor: [Parkinson disease] explode all trees (4376)

#2 (aerobic exercise or aquatic exercise or balance training or body weight support treadmill or Dance Therapy or exercise* or Exercise Movement Techniques or gait training or high-speed resistance training or hydrotherapy or multicomponent exercise program or multidisciplinary exercise program or Nordic Walking or Physiotherapy or pilates or power training or Qigong or resistance training or Robotic-assisted gait training or stretch or tai ji or Tango or treadmill training or walking or Virtual Reality or whole body vibration or Yoga) in Trials (Word variations have been searched) (155706)

#3 MeSH descriptor: [resistance training] explode all trees (3641)

#4 MeSH descriptor: [exercise] explode all trees (25628)

#5 MeSH descriptor: [tai ji] explode all trees (373)

#6 MeSH descriptor: [Qigong] explode all trees (79)

#7 MeSH descriptor: [Exercise Movement Technique] explode all trees (2215)

#8 MeSH descriptor: [Yoga] explode all trees (699)

#9 MeSH descriptor: [Virtual Reality] explode all trees (284)

#10 MeSH descriptor: [hydrotherapy] explode all trees (1575)

#11 MeSH descriptor: [Dance Therapy] explode all trees (89)

#12#2 or #3 or #4 or #5 or #6 or #7 or #8 or #9 or #10 or #11 (147882)

#13#1 and #12 (906)

## 1.6 Database: Web of Science <1965 to February 5^th^ 2024>

| # 13 | 2,403 | #12 AND #11 AND #1  Indexes=SCI-EXPANDED, SSCI, A&HCI, CPCI-S, CPCI-SSH, BKCI-S, BKCI-SSH, ESCI, CCR-EXPANDED, IC Timespan=All years |  |  |
| --- | --- | --- | --- | --- |
| # 12 | 981,618 | #10 OR #9 OR #8 OR #7 OR #6 OR #5 OR #4 OR #3 OR #2  Indexes=SCI-EXPANDED, SSCI, A&HCI, CPCI-S, CPCI-SSH, BKCI-S, BKCI-SSH, ESCI, CCR-EXPANDED, IC Timespan=All years |  |  |
| # 11 | 6,290,817 | TOPIC: ((“randomized controlled trial*” or “controlled clinical trial” or “random*” or “clinical trial*” or randomly or trial or “clinical trial” or “randomized controlled trial*” or “cross-over studies” or clinic*) )  Indexes=SCI-EXPANDED, SSCI, A&HCI, CPCI-S, CPCI-SSH, BKCI-S, BKCI-SSH, ESCI, CCR-EXPANDED, IC Timespan=All years |  |  |
| # 10 | 7,671 | TOPIC: ((Yoga or “Muscle Stretching Exercises”) )  Indexes=SCI-EXPANDED, SSCI, A&HCI, CPCI-S, CPCI-SSH, BKCI-S, BKCI-SSH, ESCI, CCR-EXPANDED, IC Timespan=All years |  |  |
| # 9 | 373 | TOPIC: ((“Dance Therapy” or “Therapy, Dance” or “Dance Therapies” or “Therapies, Dance”) )  Indexes=SCI-EXPANDED, SSCI, A&HCI, CPCI-S, CPCI-SSH, BKCI-S, BKCI-SSH, ESCI, CCR-EXPANDED, IC Timespan=All years |  |  |
| # 8 | 1,197 | TOPIC: ((hydrotherapy or Hydrotherapies or “Whirlpool Baths” or “Bath, Whirlpool” or “Baths, Whirlpool” or “Whirlpool Bath”) )  Indexes=SCI-EXPANDED, SSCI, A&HCI, CPCI-S, CPCI-SSH, BKCI-S, BKCI-SSH, ESCI, CCR-EXPANDED, IC Timespan=All years |  |  |
| # 7 | 46,480 | TOPIC: (("Virtual Reality" or "Reality, Virtual" or "Virtual Reality, Educational" or "Educational Virtual Realities" or "Educational Virtual Reality" or "Reality, Educational Virtual" or "Virtual Realities, Educational" or "Virtual Reality, Instructional" or "Instructional Virtual Realities" or "Instructional Virtual Reality" or "Realities, Instructional Virtual" or "Reality, Instructional Virtual" or "Virtual Realities, Instructional") )  Indexes=SCI-EXPANDED, SSCI, A&HCI, CPCI-S, CPCI-SSH, BKCI-S, BKCI-SSH, ESCI, CCR-EXPANDED, IC Timespan=All years |  |  |
| # 6 | 234 | TOPIC: (("Exercise Movement Techniques" or "Movement Techniques, Exercise" or "Exercise Movement Technics" or "Pilates-Based Exercises" or "Exercises, Pilates-Based" or "Pilates Based Exercises" or "Pilates Training" or "Training, Pilates"）)  Indexes=SCI-EXPANDED, SSCI, A&HCI, CPCI-S, CPCI-SSH, BKCI-S, BKCI-SSH, ESCI, CCR-EXPANDED, IC Timespan=All years |  |  |
| # 5 | 4,058 | TOPIC: (“Tai-ji” or “Tai Chi” or “Chi, Tai” or “Tai Ji Quan” or “Ji Quan, Tai” or “Quan, Tai Ji” or Taiji or Taijiquan or “T'ai Chi” or “Tai Chi Chuan” Qigong or “Qi Gong” or “Ch'i Kung”)  Indexes=SCI-EXPANDED, SSCI, A&HCI, CPCI-S, CPCI-SSH, BKCI-S, BKCI-SSH, ESCI, CCR-EXPANDED, IC Timespan=All years |  |  |
| # 4 | 625,989 | TOPIC: (Exercise* or “Exercise Program, Weight-Bearing” or “Exercise Programs, Weight-Bearing” or “Weight Bearing Exercise Program” or “Weight-Bearing Exercise Programs” Exercise* or “Physical Activity” or “Activities, Physical” or “Activity, Physical” or “Physical Activities” or “Exercise, Physical” or “Exercises, Physical” or “Physical Exercise” or “Physical Exercises” or “Exercise, Isometric” or “Exercises, Isometric” or “Isometric Exercises” or “Isometric Exercise” or “Exercise, Aerobic” or “Aerobic Exercise” or “Aerobic Exercises” or “Exercises, Aerobic” or “Exercise Training” or “Exercise Trainings” or “Training, Exercise” or “Trainings, Exercise”)  Indexes=SCI-EXPANDED, SSCI, A&HCI, CPCI-S, CPCI-SSH, BKCI-S, BKCI-SSH, ESCI, CCR-EXPANDED, IC Timespan=All years |  |  |
| # 3 | 17,738 | TOPIC: ("Resistance training” or “Training, Resistance” or “Strength Training” or “Training, Strength” or “Weight-Lifting Strengthening Program” or “Strengthening Program, Weight-Lifting” or “Strengthening Programs, Weight-Lifting” or “Weight Lifting Strengthening Program” or “Weight-Lifting Strengthening Programs” or “Weight-Lifting Exercise Program” or “Exercise Program, Weight-Lifting” or “Exercise Programs, Weight-Lifting” or “Weight Lifting Exercise Program” or “Weight-Lifting Exercise Programs” or “Weight-Bearing Strengthening Program” or “Strengthening Program, Weight-Bearing” or “Strengthening Programs, Weight-Bearing” or “Weight Bearing Strengthening Program” or “Weight-Bearing Strengthening Programs” or “Weight-Bearing Exercise Program”)  Indexes=SCI-EXPANDED, SSCI, A&HCI, CPCI-S, CPCI-SSH, BKCI-S, BKCI-SSH, ESCI, CCR-EXPANDED, IC Timespan=All years |  |  |
| # 2 | 424,147 | TOPIC: (“aerobic exercise” or “aquatic exercise” or “balance training” or “body weight support treadmill” or “Dance Therapy or exercise*” or “Exercise Movement Techniques” or “gait training” or “high-speed resistance training” or “hydrotherapy” or “multicomponent exercise program” or “multidisciplinary exercise program” or “Nordic Walking” or “Physiotherapy” or pilates or “power training” or Qigong or “resistance training” or “Robotic-assisted gait training” or stretch or “tai ji” or Tango or “treadmill training” or “walking” or “Virtual Reality” or “whole body vibration” or Yoga)  Indexes=SCI-EXPANDED, SSCI, A&HCI, CPCI-S, CPCI-SSH, BKCI-S, BKCI-SSH, ESCI, CCR-EXPANDED, IC Timespan=All years |  |  |
| # 1 | 113,262 | TOPIC: ("Idiopathic Parkinson's Disease" or "Lewy Body Parkinson's Disease" or "Parkinson's Disease, Idiopathic" or "Parkinson's Disease, Lewy Body" or "Parkinson Disease, Idiopathic" or "Parkinson's Disease" or "Idiopathic Parkinson Disease" or "Lewy Body Parkinson Disease" or "Primary Parkinsonism" or "Parkinsonism, Primary" or "Paralysis Agitans")  Indexes=SCI-EXPANDED, SSCI, A&HCI, CPCI-S, CPCI-SSH, BKCI-S, BKCI-SSH, ESCI, CCR-EXPANDED, IC Timespan=All years |  |  |

# Supplementary File 2: Definitions of exercise types and non-exercise training control

| **Abbreviation** | **Full name** | **Definitions** |
| --- | --- | --- |
| AE | Aerobic Exercise | Aerobic exercise is performed by repeating sequences of light-to-moderate intensity activities for extended periods of time.^1^ e.g., walking, bicycle, and treadmill training etc. |
| AQE | Aquatic Exercise | Gait training, balance training, resistance training, or aerobic training performed in deep or shallow water.^2^ |
| BGT | Balance and Gait Training | Single-task balance and gait training without external cues or internal and external attention |
| CON | Control group | Non-exercise intervention, usual care,^3^ or health education |
| Dance |  | Group dances other than tango, such as waltz, Irish set dancing etc. |
| MulC | Mixed Exercise Program | Two or more of the above specific types of exercise training (if it is only part of warm-up or relaxation, it is not considered as multi-mode) |
| RT | Resistance Training | Exercise training designed to improve the strength, power, endurance and size of skeletal muscles.^4^ |
| SE | Sensory Exercise | Focus on ‘internal’ or ‘external sensory’ feedback while doing balance and gait training.^5^ |
| TT | Treadmill Training Virtual Realty | Walking on a treadmill at a constant speed |
| **MBE (Mind Body Exercise)** | | |
| TC | Tai Chi | It is an internal Chinese martial art practiced for defense training, health benefits, and meditation. |
| Yoga |  | Mainly a series of methods for self-cultivation, including body-adjusting asanas (refer to yoga asana collection), breathing-adjusting breathing methods, and mind-adjusting meditation, etc., to achieve the unity of body and mind.^6^ |
| Qigong |  | It is a system of coordinated body-posture and movement, breathing, and meditation used for the purposes of health, spirituality, and martial-arts training. |

**Reference**

1. Plowman SA, Smith DL. Exercise physiology for health fitness and performance: Lippincott Williams & Wilkins; 2013.

2. Konlian C. Aquatic therapy: making a wave in the treatment of low back injuries. Orthop Nurs 1999; 18(1).

3. Goh S-L, Persson MSM, Stocks J, et al. Relative Efficacy of Different Exercises for Pain, Function, Performance and Quality of Life in Knee and Hip Osteoarthritis: Systematic Review and Network Meta-Analysis. Sports Med 2019; 49(5): 743-61.

4. Powell KE, Paluch AE, Blair SN. Physical activity for health: What kind? How much? How intense? On top of what? Annu Rev Public Health 2011; 32: 349-65.

5. Abdollahipour R, Wulf G, Psotta R, Palomo Nieto M. Performance of gymnastics skill benefits from an external focus of attention. J Sports Sci 2015; 33(17): 1807-13.

6. Cramer H, Lauche R, Haller H, Dobos G. A systematic review and meta-analysis of yoga for low back pain. Clin J Pain 2013; 29(5): 450-60.

# **Supplementary File 3: Characteristics of included studies (Table 1)**

| **Author** | **Age**  **(Mean±SD)** | **Number**  **(men)** | **Years of diagnosis** | **Hoehn and Yahr stage** | **Duration (weeks)** | **Frequency** | **Time**  **(minutes)** | **Outcome** |
| --- | --- | --- | --- | --- | --- | --- | --- | --- |
| Jo´zsef Tolla´r, MSc et al.2018 | Mul_C: 67.3±3.4 CON: 67.6±4.1 | Mul_C: 35(17) CON: 20(12) | Mul_C: 6.7±2.3 CON: 7.1±2.8 | 2-3 | 3 | 5 | 60 | TUG, time |
| Changhong Youm et al.2020 | RT: 68.0±6.8 Stretch: 72.1±6.0 | RT: 10(6) Stretch: 7(4) | RT: 6.4±3.6 Stretch: 8.0±4.0 | RT: 2.4±0.3 Stretch: 2.3±0.4 | 12 | 3 | 75 | TUG, time |
| Ariel Vieira de Morae’s Filho et al.2020 | RT: 64.7±9.0 CON: 64.4±5.8 | RT: 25(20) CON: 15(10) | RT: 5.7±4.0 CON: 7.2±7.4 | NA | 9 | 2 | 55 | TUG, time |
| Marianna Capecci et al.2014 | BGT: 66.8±4.9 CON: 68.1±5.6 | BGT: 7(4) CON: 7(4) | BGT: 9.5±7.4 CON: 9.6±4.9 | BGT: 3.3±0.7 CON: 3.3±0.9 | 4 | 3 | 40 | TUG, time |
| Nicholas P . Cherup et al.2020 | Yoga: 69.8±7.3   BGT: 71.4±12.1 | Yoga: 15(10)  BGT: 18(11) | NA | Yoga: 1.7±0.5 BGT: 2±0.8 | 12 | 2 | 45 | TUG, time |
| Madeleine E. Hackney et al.2008 | TC: 64.9± 8.3 CON: 62.6±10.2 | TC: 17(11) CON: 15(10) | TC: 8.7±4.7 CON: 5.5±3.3 | TC: 2±0.4 CON: 1.9± 0.2 | 13 | 2 | 60 | TUG, time |
| Arva Khuzema et al.2020 | TC: 72±5.22 Yoga: 68.11± 4.23 BGT: 70.89±6.01 | TC: 9(6) Yoga: 9(6) BGT: 9(7) | TC: 5.67±2.33 Yoga: 6.2±1.67 BGT: 5.23±3.12 | TC: 2.83±0.24 Yoga: 2.83±0.24 BGT: 2.78±0.25 | 8 | 5 | 35 | TUG, time |
| Jojo Y. Y. Kwok et al 2019 | Yoga: 63.7±8.2  RT: 63.5±9.3 | Yoga: 71(37)  RT: 67 (28) | NA | Yoga: 2.68 ± 0.47  RT: 2.66 ±0.54 | 8 | 3 | 85 | TUG, time |
| Ying-Yi Laio et al 2024 | Mul_C: 64.6 ± 8.6 TT: 65.1 ± 6.7 SE: 67.3 ± 7.1 | Mul_C: 12(5) TT: 12(6) SE: 12(6) | Mul_C: 6.4 ± 3.0 TT: 6.9 ± 2.8 SE: 7.9 ± 2.7 | Mul_C: 1.9 ± 0.8 TT: 2.0 ± 0.8 SE: 2.0 ± 0.7 | 6 | 2 | 60 | TUG, time |
| Xiao Lei Liu et al 2016 | Qigong: 65.84 ± 5.45 CON: 62.5 ± 3.13 | Qigong: 28(11) CON 26(14) | NA | NA | 10 | 5 | 60 | TUG, time |
| Christian Schlenstedt et al 2015 | RT: 75.7 ± 5.5 BGT: 75.7 ± 7.2 | RT:17(12) BGT: 15(9) | RT: 10.1 ± 6.0 BGT: 9.3 ± 7.9 | RT: 2.8 ± 0.26 BGT: 2.7 ± 0.4 | 7 | 2 | 60 | TUG, time |
| Pietro Santosa et al.2019 | SE: 61.7±7.3  CON: 64.5±9.8  SE: 66.6±8.2 | SE: 13(11)  CON: 14(11)  SE: 14(9) | SE: 7±2.8  CON: 6.5±2.0  SE: 7.8±3.7 | SE: 1.4±0.6  CON: 1.3±0.3  SE: 1.5±0.4 | 8 | 2 | 50 | TUG, time |
| Jooeun Song et al.2018 | SE: 68±7 CON: 65±7 | SE: 31(15) CON: 29(9) | SE: 7±4 CON: 9±6 | NA | 12 | 3 | 15 | TUG, time |
| Simon Steib, PhD et al. 2017 | SE: 67.5 ±8.2 TT: 62.5±7.9 | SE: 18(11) TT: 20(16) | SE: 7.9±4.0 TT: 7.3±4.4 | SE: 2.6±0.5 TT: 2.5±0.5 | 8 | 2 | 40 | TUG, time |
| Chun-Mei Xiao et al 2015 | CON: 66.5±2.1 Qigong: 68.1±2.3 | CON: 48(34) Qigong: 48(33) | CON: 6.2±2.6 Qigong: 5.5±3.6 | CON: 2.1±0.2 Qigong: 2.2±0.2 | 24 | 4 | 50 | TUG, time |
| Yongchang Zhuang, Med et al.2016 | 67.8±9.4 | Qigong: 49 CON: 49 | NA | NA | 24 | 4 | 60 | TUG, time |
| Wen-Chieh Yang et al.2015 | SE: 72.5±8.4 BGT: 75.4±6.3 | SE: 11(7) BGT: 12(7） | SE: 9.4±3.6 BGT: 8.3±4.1 | SE: 3±0 BGT: 3±0 | 6 | 2 | 50 | TUG, time |
| Wen-Chieh Yang et al.2019 | SE: 65.0±57.5  SE: 69.5±65.0 BGT: 66.5±55.5 | SE: 6(4)  SE: 6(4) BGT: 6(4) | SE: 5.5±2.8  SE 5.0±0.1 BGT: 3.0±0.3 | SE: 2.0±1.6  SE: 2.0±1.8 BGT: 1.5±0.9 | 4 | 3 | 30 | TUG, time |
| Tian-Yu Zhang, BS et al.2015 | TC: 66.0±11.8 Mul_C: 64.4±10.5 | TC: 20(13) Mul_C: 20(11) | TC: 6.8±5.4 Mul_C: 4.9±3.7 | TC: 2.0±0.5 Mul_C: 2.2±0.4 | 12 | 2 | 60 | TUG, time |
| Meng-Che Shih et al 2016 | SE: 67.5±10.0 BGT: 68.8±9.7 | SE: 10(9) BGT: 10(7) | SE: 4.0±3.7 BGT: 5.2±4.9 | SE: 1.6±0.8 BGT: 1.4±0.5 | 8 | 2 | 50 | TUG, time |
| Rocco Salvatore Calabro et al 2019 | SE: 70±8 TT: 73±8 | SE: 25(11) TT: 25(14) | SE: 10.0±3.0 TT: 9.3±3.0 | SE: 3.0±1.0 TT: 3.0±1.0 | 8 | 5 | 25 | TUG, time |
| Marie d EMonc Eau et al.2017 | AE: 65±8 RT: 67±10 CON: 63.3±6 | AE: 16(12) RT:15(8) CON: 15(10) | AE: 5±4.07 RT: 7±5.08 CON: 5±2.96 | AE: 1.5±1.11 RT: 2±1.11 CON: 1.5±0.74 | 12 | 2-3 | 75 | TUG, time |
| Gloria Vergara-Diaz, MD et al. 2018 | TC: 65.7±3.9 CON: 62.0±7.8 | TC: 16(9) CON: 16(7) | TC: 2.9±2.4 CON: 2.9±2.2 | TC: 2.2±0.2 CON: 2.1±0.2 | 24 | 2 | 60 | TUG, time |
| Alessandro Carvalho 2015 | AE: 64.8±11.9 RT: 64.1±9.9 BGT: 62.1±11.7 | AE: 5(4) RT: 8(6) BGT: 9(5) | AE: 6.6±1.5 RT: 6.0±2.6 BGT: 4.3±2.8 | AE: 2.6±0.5 RT: 2.1±0.6 BGT: 2.3±0.5 | 12 | 2 | 40 | TUG, time |
| Ilaria Arcolin 2016 | TT: 67.8±8.8 AE: 68.7±8.3 | TT: 13(6) AE: 16(9) | TT: 6.5±2.9 AE: 4.7±2.9 | TT: 2.3±0.5 AE: 2.3±0.5 | 3 | 5 | 60 | TUG, time |
| Paria Arfa-Fatollahkhani 2018 | CON: 61.55±8.57  TT: 60.63±9.36 | CON: 9(7) TT: 11(8) | CON: 8.50±6.34 TT: 8.89±5.14 | CON: 2.0±0.35 TT: 2.13±0.32 | 10 | 2 | 30 | TUG, time |
| Dae-Hyouk Bang 2016 | NW: 58.30±7.71 TT: 60.60±6.74 | NW: 10(5) TT: 10(4) | NW: 18.10±6.77 TT: 17.98±3.28 | NW: 2.32±0.52 TT: 2.56±0.51 | 4 | 5 | 60 | TUG, time |
| O. Bello et al.2013 | TT: 59.45±11.32  SE: 58±9.38 | TT: 11(7)  SE: 11(5) | TT: 4.82±3.28  SE: 4.95±2.59 | TT: 2.27±0.41  SE: 2.05±0.52 | 5 | 3 | 25 | TUG, time |
| Georg Ebersbach et al 2010 | Mul_C: 67.1±3.6 NW: 65.5 ±9.0 CON: 69.3 ±8.4 | Mul_C: 20(7) NW: 19(7) CON: 19(8) | Mul_C: 6.1±3.0 NW: 7.8±4.4 CON: 7.4±5.9 | Mul_C: 2.8±0.37 NW: 2.6±0.4 CON: 2.5±0.7 | 5 | 3 | 60 | TUG, time |
| Ilaria Carpinella et al 2016 | SE: 73.0±7.1 CON: 75.6±8.2 | SE: 17(14) CON: 20(9) | SE: 7.5±3.2 CON: 10.3±5.7 | SE: 12.7±0.7 CON: 2.9±0.5 | 7 | 3 | 45 | TUG, time |
| Fang-Yu Cheng et al 2017 | TT: 65.8 ± 11.5 CON: 67.3 ± 6.4 | TT: 12(9) CON: 12(8) | TT: 6.1 ± 4.1 CON: 8.1 ± 4.6 | TT: 1.8 ± 0.6 CON: 2.0 ± 0.8 | 5 | 2.5 | 40 | TUG, time |
| Silvia Rios Romenets et al.2015 | CON: 64.3±8.1 Tango: 63.2±9.9 | CON: 15(7) Tango: 18(12) | CON 7.7±4.6 Tango: 5.5±4.4 | CON: 2.0±0.5 Tango: 1.7±0.6 | 12 | 2 | 60 | TUG, time |
| Adriana Costa-Ribeiro et al.2016 | SE: 61.1±9.1  SE: 62.0±16.7 | SE: 11(8)  SE: 11(7) | SE: 2.4±0.7  SE: 2.3±0.4 | SE: 6.1±3.8  SE: 6.3±3.7 | 4 | 3 | 43 | TUG, time |
| Lucia Cugusi et al.2015 | NW: 68.1 ± 8.7 CON: 66.6±7.3 | NW: 10 (8) CON: 10(8) | NW: 7±2 CON: 7±4 | NW: 2.4 ± 0.8 CON: 2.3 ± 0.5 | 12 | 2 | 60 | TUG, time |
| Tiago Alencar de Lima et al.2019 | CON: 67.2 ± 5.2 RT: 66.2 ± 5.5 | CON: 16(NA) RT: 17(NA) | NA | CON: 1.93±0.80 RT: 2.07±0.80 | 20 | 2 | 35 | TUG, time |
| G.Frazzitta et al.2007 | SE: 66.6±10.0  AE: 65.0±8.8 | SE: 30(13) AE: 30(17) | NA | SE: 2.8 ±0.4 AE: 2.8±0.4 | 4 | 6 | 35 | TUG, time |
| Victoria A Goodwin et al.2009 | Mul_C: 72.0±8.6 CON: 70.1±8.3 | Mul_C: 64(39) CON: 66(35) | Mul_C: 9.1±6.4 CON 8.2±6.4 | Mul_C: 2.6 ±0.9 CON: 2.4 ±0.9 | 10 | 1 | 60 | TUG, time |
| Madeleine E. Hackney et al. 2015 | Tango: 72.6±2.2 RT: 69.6±2.1 | Tango: 9(6) RT: 10(6) | Tango: 6.2±1.5 RT: 3.3±0.5 | Tango: 2.3±0.7 RT: 2.2±0.6 | 13 | 2 | 60 | TUG, time |
| Ryan P. Hubble et al.2017 | Mul_C: 67.5±5.8 CON: 63.3±4.9 | Mul_C: 11(8) CON: 11(7) | Mul_C: 7.0 ±5.0 CON: 6.5±5.2 | Mul_C: 2.0±0.7  CON: 1.8±0.6 | 12 | 1 | 90 | TUG, time |
| Adriano Zanardi da Silva 2018 | AQE: 63.12 ± 13.61 CON: 64.23 ± 13.45 | AQE: 14(6) CON: 11(5) | NA | AUR: 3±1 CON: 3±1 | 10 | 2 | 60 | TUG, time |
| Daniele Volpe et al.2014 | SE: 66.5±10.4 BGT: 69.5±6.5 | SE: 20(7) BGT: 20(9) | SE: 6.0±5.0 BGT: 6.5±3.7 | SE: 3.0±0.0 BGT: 3.0±0.7 | 8 | 5 | 60 | TUG, time |
| Michael D. Sage et al.2009 | SE: 64.2±10.3 AE: 65.1±9.3 CON: 68.6±8.7 | SE: 18(12) AE: 13(6) CON: 15(7) | SE: 4.7±4.9 AE: 3.2±2.9  CON: 2.5±2.2 | NA | 12 | 3 | 50 | TUG, time |
| Zahra Kadivar 2011 | SE: 73.3±2.2 BGT: 70.5±2.2 | SE: 8(5) BGT: 8(6) | SE: 8.9±1.8 BGT: 7.5±1.2 | SE: 2.69±0.56 BGT: 2.69±0.56 | 6 | 3 | 50 | TUG, time |
| Paolo Solla 2019 | Dance: 67.8±5.9 CON: 67.1±6.3 | Dance: 10(6) CON: 10(7) | Dance: 4.4±4.5 CON: 5.0±2.9 | Dance: 2.1±0.6 CON: 2.3±0.4 | 12 | 2 | 90 | TUG, time |
| Kristi Michels 2018 | Tango: 66.44±NA CON: 75.50±NA | Tango: 9(NA) CON: 4(NA) | NA | Tango: 2.11±0.33 CON: 2.50±1.00 | 10 | 2 | 60 | TUG, time |
| D Kunkel 2017 | Dance: 71.3±7.7 CON: 69.7±6.0 | Dance: 36(19) CON: 15(6) | Dance: 4.7±3.5 CON: 7.0±4.9 | Dance: 2.11±0.84 CON: 2.13±0.72 | 10 | 2 | 60 | TUG, time |
| Lisa M. Shulman 2012 | TT: 66.1±9.7 TT: 65.8±11.5 RT: 65.3±11.3 | TT: 23(16) TT: 22(16) RT: 22(18) | TT: 5.9±3.9 TT: 6.3±3.5 RT: 6.3±4.0 | TT: 2.15±0.34 TT: 2.16±0.35 RT: 2.23±0.39 | 12 | 3 | 45 | TUG, time |
| Petra Pohl et al.2015 | 68.2±5.1 | Dance: 12(NA) CON: 6(NA) | 8.8±3.8 | 2.4±0.7 | 6 | 2 | 60 | TUG, time |
| Gloria Vergara-Diaz, MD et al. 2018 | TC: 65.7±3.9 CON: 62.0±7.8 | TC: 16(9) CON: 16(7) | TC: 2.9±2.4 CON: 2.9±2.2 | TC: 2.2±0.2 CON: 2.1±0.2 | 24 | 2 | 60 | TUG, time |
| Emine Eda Kurt et al 2017 | AQE: 62.41 ± 6.76 Mul_C: 63.61 ± 7.18 | AQE: 20(11) Mul_C: 20(13) | NA | AQE: 2.37±0.39 Mul_C: 2.32±0.40 | 5 | 5 | 60 | TUG, time |
| Yesim Kurtais et al 2008 | TT: 63.8±10.6 CON: 65.7±5.3 | TT: 12(5) CON: 12(7) | TT: 5.3±0.8 CON: 5.4±1.2 | TT: 2.5±0.7 CON: 2.2±0.8 | 6 | 3 | 40 | TUG, time |
| Leon CP Leal et al 2019 | CON: 64.9±2.32  RT: 65.2±2.05 | CON: 27(13)  RT: 27(14) | NA | CON: 2±0.5  RT: 2±0.5 | 24 | 2 | 32.5 | TUG, time |
| E.P. Monteiro et al 2016 | NW: 64.9±10.2 BGT: 70.5±5.8 | NW: 16(13) BGT: 17(7) | NW: 5.5±3.3 BGT: 5.09±4.1 | NW: 1.5±0.5 BGT: 2.0±1.0 | 6 | 2 | 60 | TUG, time |
| Meg E. Morris et al 2015 | RT: 67.4±10.4  SE: 68.4±9.9 CON: 67.9±8.4 | RT: 70(42)  SE 69(46) CON: 71(52) | RT: 7.2±6.2  SE: 6±5.5 CON: 6.9±5.2 | RT: 2.39±0.77  SE: 2.40±0.81 CON: 2.61±0.90 | 8 | 1 | 120 | TUG, time |
| Grazia Palamara et al.2017 | AQE: 70.9±5.7 Mul_C: 70.8±5.3 | AQE: 17(9) Mul_C: 17(11) | NA | AQE: 2.8±0.5 Mul_C: 3.1±0.2 | 4 | 4 | 60 | TUG, time |
| A. Park et al 2013 | RT: 60.1±6.6 CON: 59.8±6.3 | RT: 15(10) CON: 16(10) | NA | NA | 48 | 3 | 60 | TUG, time |
| Ellen Poliakoff et al 2013 | Mul_C: 68.8±7.3 CON: 66.6±7.3 | Mul_C: 12(9) CON: 10(8) | Mul_C: 7.9±3.0 CON: 4.6±3.9 | NA | 10 | 1 | 60 | TUG, time |
| Cornelia Schlick et al 2015 | SE: 71.2±10.9  TT: 68.9±6.8 | SE: 10(2) TT: 10(4) | SE: 10.4±5.2 TT: 9.1±3.1 | SE: 2.8±0.9 TT: 2.7±0.7 | 5 | 2.5 | 35 | TUG, time |
| CARLA SILVA-BATISTA et al 2016 | CON: 64.2±8.3  RT: 64.1±9.1  RT: 64.2±10.6 | CON: 13(9)  RT: 13(10)  RT: 13(10) | CON: 10.7±6.1  RT: 9.6±3.9  RT: 10.5±4.1 | CON: 2.5±0.4  RT: 2.5±0.5  RT: 2.5±0.4 | 12 | 2 | 60 | TUG, time |
| Carla Silva-Batista et al. 2016 | CON: 64.2±8.3  RT: 64.2±10.6 | CON: 13(9)  RT: 13(10) | CON: 10.7±6.1  RT: 10.5±4.1 | CON: 2.5±0.4  RT: 2.5±0.4 | 12 | 2 | 60 | TUG, time |
| Nicolien M van der Kolk et al.2017 | NA | AE: 22(NA) CON: 15(NA) | NA | NA | 24 | 3 | 30 | TUG, time |
| Daniele Volpe1 et al.2014 | AQE: 68 ± 7 BGT: 66 ± 8 | AQE:17(NA) BGT:17(NA) | AQE: 7.5 ± 5.1 BGT: 7.6 ± 4.63 | AQE: 2.82 ± 0.3 BGT: 2.65 ± 0.49 | 8 | 5 | 60 | TUG, time |
| Daniele Volpe1 et al.2016 | AQE: 70.6 ± 7.8  Mul_C: 70 ± 7.8 | AQE: 15(9) Mul_C: 15(10) | AQE: 9.4 ± 7.5  Mul_C: 9 ± 7.0 | AQE: 2.6 ± 0.5 Mul_C: 2.7 ± 0.5 | 8 | 5 | 60 | TUG, time |
| Meg E. Morris al.2009 | SE: 72.5±5.8 Mul_C: 73.5±5.7 | SE: 14(NA) Mul_C: 14(NA) | NA | NA | 2 | 8 | 40 | TUG, time |
| Helgerud J et al.2020 | RT: 72±8.0 CON: 62.0±11 | RT: 15(7) CON: 7(2) | RT: 8.8±4.9 CON: 7.3±2.5 | RT: 2.3±0.1 CON: 2.7±0.7 | 4 | 3 | 60 | TUG, time |
| José Ma Cancela 2019 | AQE: 67.7±4.6 Mul_C: 69.2±4.4 | AQE: 7(6) Mul_C: 5(3) | NA | AQE: 2.3±0.8 Mul_C: 2.2±0.5 | 8 | 3 | 50 | TUG, time |
| Tamine T.C. Capato et al.2020 | SE: 74±8 BGT: 67±13 CON: 73±10 | SE: 56(27) BGT: 50(32) CON: 48(29) | SE: 5±5.2 BGT: 6±5.9 CON: 8±9.6 | SE: 2.3±0.8 BGT: 2.2±0.8 CON: 2.3±0.7 | 5 | 2 | 45 | TUG, time |
| Tamine T.C. Capato et al.2020 | SE: 77±7 BGT: 78±10 | SE: 17(9) BGT: 18(12) | SE: 17±9 BGT: 11±4 | NA | 5 | 2 | 45 | TUG, time |
| Qiang Gao et al.2014 | TC: 69.5±7.3 CON: 68.3±8.5 | TC: 37(23) CON: 39(27) | TC: 9.2±8.6 CON: 8.4±8.2 | TC: 2.4±0.5 CON: 2.4±0.7 | 12 | 3 | 60 | TUG, time |
| A Nieuwboer et al.2014 | SE: 67.5±7.8 CON: 69±7.8 | SE: 76(48) CON: 77(40) | SE: 7±5.2 CON: 8±5.9 | SE: 2.6±0.7 CON: 2.7±0.7 | 3 | 3 | 30 | TUG, time |
| Hsin- Hsuan Liu, PT et al.2022 | BGT: 70.93±7.23 CON: 64.79±5.86 | BGT: 14(8) CON: 14(8) | BGT: 6.82 ± 3.94 CON: 6.96 ± 6.27 | BGT: 1.96 ± 0.72 CON: 1.54 ± 0.72 | 8 | 2 | 60 | TUG, time |
| Zhenlan Li et al.2022 | Wu Qin Xi：67.57±3.95 CON: 70±5.59 | Wu Qin Xi：20(13)  CON : 20(16) | Wu Qin Xi：6.83 ± 4.09  CON :7.76 ± 4.55 | Wu Qin Xi：1.5±1.5  CON : 1.6±0.59 | 8 | 2 | 60 | TUG, time |

N/A not available, AE Aerobic Exercise, AQE Aquatic Exercise, BGT Balance and Gait Training,CON Control group, MulC Multicomponent Exercise Program, RT Resistance Training, TT Treadmil traing, TC Tai Chi, SE Sensory Exercise, TUG Time Up Go.

**Inclusion of study-specific data (Table 2)**

| **Author** | **Study** | **agent** | **dose** | **y** | **SE** | **n** | **MET** | **residual dose** | **MET-min/week** | **total MET** | **Time** | **Frequency** | **Period** |
| --- | --- | --- | --- | --- | --- | --- | --- | --- | --- | --- | --- | --- | --- |
| Josef et al.2018 | 1 | CON | 0 | -0.40 | 0.917606 | 20 | 0 | 0 | 0 | 0 | 0 | 0 | 0 |
|  | 1 | MulC | 750 | -6.20 | 0.560357 | 35 | 4.3 | 110 | 860 | 2580 | 40 | 5 | 3 |
| Changhong Youm et al.2020 | 2 | CON | 0 | 0.20 | 0.415761 | 7 | 0 | 0 | 0 | 0 | 60 | 3 | 12 |
|  | 2 | RT | 750 | -2.00 | 0.496991 | 10 | 3.5 | -120 | 630 | 7560 | 60 | 3 | 12 |
| Ariel Vieira de Morae’s Filho et al.2020 | 3 | CON | 0 | 0.70 | 0.819199 | 15 | 0 | 0 | 0 | 0 | 0 | 0 | 0 |
|  | 3 | RT | 500 | -1.80 | 0.458258 | 25 | 3.5 | -115 | 385 | 3465 | 55 | 2 | 9 |
| Marianna Capecci et al.2014 | 4 | CON | 0 | 0.00 | 3.363884 | 7 | 0 | 0 | 0 | 0 | 0 | 0 | 0 |
|  | 4 | BGT | 500 | -1.00 | 3.478678 | 7 | 3.8 | -44 | 456 | 1824 | 40 | 3 | 4 |
| Nicholas P. Cherup et al.2020 | 5 | BGT | 250 | -1.00 | 0.968102 | 18 | 3.8 | 92 | 342 | 4104 | 45 | 2 | 12 |
|  | 5 | MBE | 250 | -0.90 | 0.626099 | 15 | 3 | 20 | 270 | 3240 | 45 | 2 | 12 |
| Madeleine E. Hackney et al.2008 | 6 | CON | 0 | -0.10 | 0.305085 | 13 | 0 | 0 | 0 | 0 | 0 | 0 | 0 |
|  | 6 | MBE | 250 | -1.00 | 0.027735 | 13 | 3 | 110 | 360 | 4680 | 60 | 2 | 13 |
| Arva Khuzema et al.2020 | 7 | MBE | 500 | -3.33 | 1.660793 | 9 | 3 | 25 | 525 | 4200 | 35 | 5 | 8 |
|  | 7 | BGT | 750 | -1.38 | 2.288435 | 9 | 3.8 | -85 | 665 | 5320 | 35 | 5 | 8 |
|  | 7 | MBE | 500 | -1.39 | 4.454546 | 9 | 3 | 25 | 525 | 4200 | 35 | 5 | 8 |
| Jojo Y. Y. Kwok et al 2019 | 8 | RT | 750 | -1.64 | 0.685038 | 67 | 3.5 | -120 | 630 | 5040 | 60 | 3 | 8 |
|  | 8 | MBE | 750 | -2.82 | 1.826927 | 71 | 3 | -210 | 540 | 4320 | 60 | 3 | 8 |
| Ying-Yi Laio et al 2024 | 9 | CON | 0 | 0.70 | 0.490748 | 12 | 0 | 0 | 0 | 0 | 0 | 0 | 0 |
|  | 9 | MulC | 500 | -1.10 | 0.028868 | 12 | 3.5 | -80 | 420 | 2520 | 60 | 2 | 6 |
|  | 9 | SE | 500 | -2.90 | 0.635085 | 12 | 3.8 | -44 | 456 | 2736 | 60 | 2 | 6 |
| Xiao Lei Liu et al 2016 | 10 | CON | 0 | -0.24 | 0.681962 | 18 | 0 | 0 | 0 | 0 | 0 | 0 | 0 |
|  | 10 | MBE | 500 | -4.79 | 0.534493 | 23 | 3 | 100 | 600 | 6000 | 40 | 5 | 10 |
| Schlenstedt et al 2015 | 11 | RT | 500 | -1.70 | 0.69958 | 17 | 3.5 | -80 | 420 | 2940 | 60 | 2 | 7 |
|  | 11 | BGT | 250 | -0.20 | 0.675278 | 15 | 2.3 | 26 | 276 | 1932 | 60 | 2 | 7 |
| Pietro Santosa et al.2019 | 12 | CON | 0 | -2.50 | 0.853982 | 14 | 0 | 0 | 0 | 0 | 50 | 2 | 8 |
|  | 12 | SE | 500 | -3.00 | 1.231322 | 13 | 3.8 | -120 | 380 | 3040 | 50 | 2 | 8 |
| Pietro Santosa et al.2019 | 12 | CON | 0 | -2.50 | 0.853982 | 14 | 0 | 0 | 0 | 0 | 50 | 2 | 8 |
|  | 12 | SE | 500 | -3.30 | 0.668153 | 14 | 3.8 | -120 | 380 | 3040 | 50 | 2 | 8 |
| Jooeun Song et al.2018 | 13 | CON | 0 | -0.50 | 0.308 | 25 | 0 | 0 | 0 | 0 | 0 | 0 | 0 |
|  | 13 | SE | 250 | 0.15 | 0.228669 | 28 | 3.8 | -79 | 171 | 2052 | 15 | 3 | 12 |
| Simon Steib, PhD et al. 2017 | 14 | TT | 250 | 0.30 | 0.268328 | 20 | 2.5 | -50 | 200 | 1600 | 40 | 2 | 8 |
|  | 14 | SE | 250 | -0.80 | 0.377124 | 18 | 3.8 | 54 | 304 | 2432 | 40 | 2 | 8 |
| Chun-Mei Xiao et al 2015 | 15 | CON | 0 | 1.00 | 2.687936 | 44 | 0 | 0 | 0 | 0 | 0 | 0 | 0 |
|  | 15 | MBE | 500 | -1.20 | 1.735504 | 45 | 3 | 100 | 600 | 14400 | 50 | 4 | 24 |
| Yongchang Zhuang, Med et al.2016 | 16 | CON | 0 | -0.90 | 0.290084 | 33 | 0 | 0 | 0 | 0 | 60 | 4 | 24 |
|  | 16 | MBE | 750 | -1.00 | 0.45269 | 35 | 3 | -30 | 720 | 17280 | 60 | 4 | 24 |
| Wen-Chieh Yang et al.2015 | 17 | BGT | 500 | -3.10 | 3.231615 | 12 | 3.8 | -120 | 380 | 2280 | 50 | 2 | 6 |
|  | 17 | SE | 500 | -3.30 | 3.27428 | 11 | 3.8 | -120 | 380 | 2280 | 50 | 2 | 6 |
| Wen-Chieh Yang et al.2019 | 17 | SE | 250 | -1.20 | 0.628702 | 6 | 3.8 | 92 | 342 | 1368 | 30 | 3 | 4 |
|  | 17 | BGT | 250 | 0.30 | 1.931722 | 6 | 3.8 | 92 | 342 | 1368 | 30 | 3 | 4 |
| Wen-Chieh Yang et al.2019 | 17 | SE | 250 | -2.90 | 2.148724 | 6 | 3.8 | 92 | 342 | 1368 | 30 | 3 | 4 |
|  | 17 | BGT | 250 | 0.30 | 1.931722 | 6 | 3.8 | 92 | 342 | 1368 | 30 | 3 | 4 |
| Tian-Yu Zhang, BS et al.2015 | 18 | MulC | 500 | -2.13 | 0.406964 | 20 | 4.75 | 70 | 570 | 6840 | 60 | 2 | 12 |
|  | 18 | MBE | 250 | -1.91 | 0.72225 | 20 | 3 | 110 | 360 | 4320 | 60 | 2 | 12 |
| Meng-Che Shih et al 2016 | 19 | BGT | 500 | -0.87 | 1.321923 | 10 | 3.8 | -120 | 380 | 3040 | 50 | 2 | 8 |
|  | 19 | SE | 500 | -0.79 | 0.695162 | 10 | 3.8 | -120 | 380 | 3040 | 50 | 2 | 8 |
| Rocco Salvatore et al 2019 | 20 | SE | 500 | -2.00 | 1.637071 | 25 | 3 | -125 | 375 | 3000 | 25 | 5 | 8 |
|  | 20 | TT | 250 | -1.00 | 1.4 | 25 | 2.3 | 37.5 | 287.5 | 2300 | 25 | 5 | 8 |
| Marie et al.2017 | 21 | CON | 0 | 0.00 | 0.05164 | 15 | 0 | 0 | 0 | 0 | 0 | 0 | 0 |
|  | 21 | RT | 1200 | -0.10 | 0.093095 | 15 | 5.8 | 105 | 1305 | 15660 | 75 | 3 | 12 |
|  | 21 | AE | 750 | -0.10 | 0.066144 | 16 | 3.5 | 37.5 | 787.5 | 9450 | 75 | 3 | 12 |
| Gloria et al. 2018 | 22 | CON | 0 | 0.00 | 0.494874 | 13 | 0 | 0 | 0 | 0 | 0 | 0 | 0 |
|  | 22 | MBE | 250 | -0.58 | 0.701463 | 14 | 3 | 110 | 360 | 8640 | 60 | 2 | 24 |
| Alessandro Carvalho et.al.2015 | 23 | RT | 250 | -2.50 | 0.863858 | 8 | 4 | 70 | 320 | 3840 | 40 | 2 | 12 |
|  | 23 | BGT | 250 | -2.40 | 0.635959 | 9 | 3.8 | 54 | 304 | 3648 | 40 | 2 | 12 |
|  | 23 | AE | 250 | -3.10 | 1.323631 | 5 | 4.3 | 94 | 344 | 4128 | 40 | 2 | 12 |
| Ilaria et.al. 2016 | 24 | TT | 500 | -1.60 | 0.402874 | 13 | 2.3 | -40 | 460 | 1380 | 40 | 5 | 3 |
|  | 24 | AE | 750 | -0.70 | 0.463006 | 16 | 3.5 | -50 | 700 | 2100 | 40 | 5 | 3 |
| Paria et.al. 2018 | 25 | CON | 0 | -2.56 | 3.72812 | 9 | 0 | 0 | 0 | 0 | 0 | 0 | 0 |
|  | 25 | TT | 250 | -3.92 | 2.305036 | 11 | 2.3 | -112 | 138 | 1380 | 30 | 2 | 10 |
| Dae-Hyouk Bang 2016 | 26 | TT | 500 | -3.48 | 0.617746 | 10 | 2.3 | 75 | 575 | 2300 | 50 | 5 | 4 |
|  | 26 | AE | 750 | -4.72 | 0.632052 | 10 | 3.5 | 125 | 875 | 3500 | 50 | 5 | 4 |
| O. Bello et al.2013 | 27 | SE | 250 | -1.61 | 0.455941 | 11 | 3.5 | 12.5 | 262.5 | 1312.5 | 25 | 3 | 5 |
|  | 27 | TT | 250 | 0.13 | 0.18063 | 11 | 2.3 | -77.5 | 172.5 | 862.5 | 25 | 3 | 5 |
| Georg Ebersbach et al 2010 | 28 | CON | 0 | 0.44 | 0.277593 | 19 | 0 | 0 | 0 | 0 | 0 | 0 | 0 |
|  | 28 | MulC | 750 | -0.75 | 0.433797 | 20 | 4.75 | 105 | 855 | 4275 | 60 | 3 | 5 |
|  | 28 | AE | 750 | 0.58 | 0.394595 | 19 | 4.8 | 114 | 864 | 4320 | 60 | 3 | 5 |
| Ilaria Carpinella et al 2016 | 29 | CON | 0 | 0.40 | 3.848961 | 20 | 0 | 0 | 0 | 0 | 0 | 0 | 0 |
|  | 29 | SE | 250 | -1.00 | 1.592076 | 17 | 2.3 | 60.5 | 310.5 | 2173.5 | 45 | 3 | 7 |
| Fang-Yu Cheng et al 2017 | 30 | CON | 0 | -0.50 | 0.78475 | 12 | 0 | 0 | 0 | 0 | 0 | 0 | 0 |
|  | 30 | TT | 250 | -2.10 | 1.123981 | 12 | 2.3 | -20 | 230 | 1150 | 40 | 2.5 | 5 |
| Silvia Rios Romenets et al.2015 | 31 | CON | 0 | 0.10 | 0.610464 | 15 | 0 | 0 | 0 | 0 | 0 | 0 | 0 |
|  | 31 | Dance | 250 | -1.30 | 0.424918 | 18 | 3 | 110 | 360 | 4320 | 60 | 2 | 12 |
| Adriana Costa-Ribeiro et al.2016 | 32 | SE | 250 | -3.00 | 1.266348 | 11 | 2.8 | 111.2 | 361.2 | 1444.8 | 43 | 3 | 4 |
|  | 32 | SE | 500 | -2.30 | 0.54272 | 11 | 4.8 | 119.2 | 619.2 | 2476.8 | 43 | 3 | 4 |
| Lucia Cugusi et al.2015 | 33 | CON | 0 | 0.90 | 0.727324 | 10 | 0 | 0 | 0 | 0 | 0 | 0 | 0 |
|  | 33 | AE | 500 | -0.70 | 0.782943 | 10 | 4.8 | 76 | 576 | 6912 | 60 | 2 | 12 |
| Tiago Alencar de Lima et al.2019 | 34 | CON | 0 | 1.40 | 1.605265 | 16 | 0 | 0 | 0 | 0 | 0 | 0 | 0 |
|  | 34 | RT | 250 | -6.80 | 1.666098 | 17 | 3.5 | -5 | 245 | 4900 | 35 | 2 | 20 |
| Tiago Alencar de Lima et al.2019 | 34 | CON | 0 | 0.90 | 0.33541 | 20 | 0 | 0 | 0 | 0 | 0 | 0 | 0 |
|  | 34 | RT | 250 | -1.10 | 0.435316 | 20 | 3.5 | -5 | 245 | 4900 | 35 | 2 | 20 |
| G.Frazzitta et al.2007 | 35 | SE | 750 | -3.30 | 1.22692 | 30 | 3.5 | -15 | 735 | 2940 | 35 | 6 | 4 |
|  | 35 | AE | 750 | -3.00 | 0.689202 | 30 | 3.5 | -15 | 735 | 2940 | 35 | 6 | 4 |
| Victoria A Goodwin et al.2009 | 36 | CON | 0 | 1.60 | 1.162373 | 63 | 0 | 0 | 0 | 0 | 0 | 0 | 0 |
|  | 36 | MulC | 250 | 0.30 | 0.938639 | 61 | 4.75 | 35 | 285 | 2850 | 60 | 1 | 10 |
| Madeleine E. Hackney et al. 2015 | 37 | RT | 500 | 0.10 | 0.398447 | 10 | 3.5 | -80 | 420 | 5460 | 60 | 2 | 13 |
|  | 37 | Dance | 250 | -0.90 | 0.4 | 9 | 3 | 110 | 360 | 4680 | 60 | 2 | 13 |
| Ryan P. Hubble et al.2017 | 38 | CON | 0 | -0.34 | 0.53066 | 11 | 0 | 0 | 0 | 0 | 0 | 0 | 0 |
|  | 38 | MulC | 500 | 0.11 | 0.53066 | 11 | 4.75 | -72.5 | 427.5 | 5130 | 90 | 1 | 12 |
| Adriano da Silva 2018 | 39 | CON | 0 | 1.25 | 1.162329 | 11 | 0 | 0 | 0 | 0 | 0 | 0 | 0 |
|  | 39 | AQE | 750 | -2.52 | 1.023057 | 14 | 5.3 | -114 | 636 | 6360 | 60 | 2 | 10 |
| Daniele Volpe et al.2014 | 40 | BGT | 750 | -0.90 | 0.623298 | 20 | 2.3 | -60 | 690 | 5520 | 60 | 5 | 8 |
|  | 40 | SE | 750 | -2.20 | 0.998248 | 20 | 2.3 | -60 | 690 | 5520 | 60 | 5 | 8 |
| Michael D. Sage et al.2009 | 41 | CON | 0 | 0.00 | 0.600555 | 15 | 0 | 0 | 0 | 0 | 0 | 0 | 0 |
|  | 41 | SE | 500 | -0.50 | 0.487055 | 18 | 3 | -50 | 450 | 5400 | 50 | 3 | 12 |
|  | 41 | AE | 500 | -0.70 | 0.667371 | 13 | 3.5 | 25 | 525 | 6300 | 50 | 3 | 12 |
| Zahra Kadivar 2011 | 42 | BGT | 500 | -2.62 | 0.848528 | 8 | 2.5 | -125 | 375 | 2250 | 50 | 3 | 6 |
|  | 42 | SE | 250 | -5.70 | 0.777817 | 8 | 2.3 | 95 | 345 | 2070 | 50 | 3 | 6 |
| Paolo Solla 2019 | 42 | CON | 0 | -0.48 | 0.287412 | 17 | 0 | 0 | 0 | 0 | 0 | 0 | 0 |
|  | 42 | Dance | 500 | -1.82 | 0.234361 | 16 | 3 | 40 | 540 | 6480 | 90 | 2 | 12 |
| Kristi Michels 2018 | 43 | CON | 0 | -0.31 | 4.789616 | 4 | 0 | 0 | 0 | 0 | 0 | 0 | 0 |
|  | 43 | Dance | 250 | -0.57 | 0.531863 | 9 | 3 | 110 | 360 | 3600 | 60 | 2 | 10 |
| D Kunkel 2017 | 44 | CON | 0 | -1.30 | 1.478513 | 15 | 0 | 0 | 0 | 0 | 0 | 0 | 0 |
|  | 44 | Dance | 750 | 0.70 | 0.952416 | 31 | 5.4 | -102 | 648 | 6480 | 60 | 2 | 10 |
| Lisa M. Shulman 2012 | 45 | RT | 500 | 0.50 | 1.323564 | 22 | 3.5 | -27.5 | 472.5 | 5670 | 45 | 3 | 12 |
|  | 45 | TT | 750 | -0.40 | 0.75564 | 23 | 6 | 60 | 810 | 9720 | 45 | 3 | 12 |
| Lisa M. Shulman 2012 | 45 | RT | 500 | 0.50 | 1.323564 | 22 | 3.5 | -27.5 | 472.5 | 5670 | 45 | 3 | 12 |
|  | 45 | TT | 750 | -0.50 | 0.700479 | 21 | 6 | 60 | 810 | 9720 | 45 | 3 | 12 |
| Lisa M. Shulman 2015 | 46 | TT | 250 | 1.10 | 1.48181 | 59 | 2.3 | 60.5 | 310.5 | 1863 | 45 | 3 | 6 |
|  | 46 | SE | 500 | -0.64 | 1.482903 | 62 | 3.8 | 13 | 513 | 3078 | 45 | 3 | 6 |
| Petra Pohl et al.2015 | 47 | CON | 0 | 1.00 | 1.057363 | 6 | 0 | 0 | 0 | 0 | 0 | 0 | 0 |
|  | 47 | Dance | 750 | -0.50 | 1.174908 | 12 | 5.4 | -102 | 648 | 3888 | 60 | 2 | 6 |
| Diaz, MD et al. 2018 | 48 | CON | 0 | -0.49 | 0.461303 | 13 | 0 | 0 | 0 | 0 | 0 | 0 | 0 |
|  | 48 | MBE | 250 | -0.49 | 0.682333 | 14 | 3 | 110 | 360 | 8640 | 60 | 2 | 24 |
| Eda Kurt et al 2017 | 49 | MulC | 1000 | -0.85 | 1.510464 | 20 | 4.15 | 37.5 | 1037.5 | 5187.5 | 50 | 5 | 5 |
|  | 49 | AQE | 1500 | -5.01 | 1.218325 | 20 | 4.5 | -150 | 1350 | 6750 | 60 | 5 | 5 |
| Kurtais et al 2008 | 50 | CON | 0 | -1.50 | 0.728583 | 12 | 0 | 0 | 0 | 0 | 0 | 0 | 0 |
|  | 50 | TT | 250 | -2.70 | 1.879051 | 12 | 2.3 | 26 | 276 | 0 | 40 | 3 |  |
| Leon CP Leal et al 2019 | 51 | CON | 0 | 0.70 | 0.837324 | 27 | 0 | 0 | 0 | 0 | 0 | 0 | 0 |
|  | 51 | RT | 250 | -3.10 | 0.800231 | 27 | 5 | 75 | 325 | 7800 | 32.5 | 2 | 24 |
| E.P. Monteiro et al 2016 | 52 | BGT | 500 | -2.78 | 0.557832 | 17 | 3.8 | -44 | 456 | 2736 | 60 | 2 | 6 |
|  | 52 | AE | 500 | 1.42 | 0.1 | 16 | 4.8 | 76 | 576 | 3456 | 60 | 2 | 6 |
| Meg E. Morris et al 2015 | 53 | CON | 0 | 1.10 | 0.749152 | 57 | 0 | 0 | 0 | 0 | 0 | 0 | 0 |
|  | 53 | RT | 500 | 0.00 | 0.752012 | 67 | 3.5 | -80 | 420 | 2520 | 60 | 2 | 6 |
|  | 53 | SE | 500 | 0.80 | 0.663782 | 66 | 3.8 | -44 | 456 | 2736 | 60 | 2 | 6 |
| Grazia Palamara et al.2017 | 54 | MulC | 1000 | -2.40 | 0.584934 | 17 | 4.15 | -4 | 996 | 3984 | 60 | 4 | 4 |
|  | 54 | AQE | 1200 | -3.40 | 0.708934 | 17 | 5.3 | 72 | 1272 | 5088 | 60 | 4 | 4 |
| A. Park et al 2013 | 55 | CON | 0 | -0.17 | 0.487987 | 16 | 0 | 0 | 0 | 0 | 0 | 0 | 0 |
|  | 55 | RT | 750 | -0.75 | 0.441369 | 15 | 3.5 | -120 | 630 | 30240 | 60 | 3 | 48 |
| Ellen Poliakoff et al 2013 | 56 | CON | 0 | -0.20 | 0.798123 | 10 | 0 | 0 | 0 | 0 | 0 | 0 | 0 |
|  | 56 | MulC | 250 | -0.30 | 0.996813 | 11 | 4.75 | 35 | 285 | 2850 | 60 | 1 | 10 |
| Cornelia Schlick et al 2015 | 57 | SE | 250 | -0.10 | 1.388884 | 10 | 3 | 12.5 | 262.5 | 1312.5 | 35 | 2.5 | 5 |
|  | 57 | TT | 250 | -2.60 | 1.977119 | 10 | 2.3 | -48.75 | 201.25 | 1006.25 | 35 | 2.5 | 5 |
| CARLA SILVA-BATISTA et al 2016 | 58 | CON | 0 | 1.10 | 0.526965 | 13 | 0 | 0 | 0 | 0 | 0 | 0 | 0 |
|  | 58 | RT | 500 | -1.90 | 0.66564 | 13 | 3.5 | -80 | 420 | 5040 | 60 | 2 | 12 |
| CARLA SILVA-BATISTA et al 2016 | 59 | CON | 0 | 1.10 | 0.526965 | 13 | 0 | 0 | 0 | 0 | 0 | 0 | 0 |
|  | 59 | RT | 500 | -0.70 | 0.582435 | 13 | 3.5 | -80 | 420 | 5040 | 60 | 2 | 12 |
| Carla Silva-Batista et al. 2017 | 60 | CON | 0 | 1.10 | 0.526965 | 13 | 0 | 0 | 0 | 0 | 0 | 0 | 0 |
|  | 60 | RT | 500 | -1.90 | 0.66564 | 13 | 3.5 | -80 | 420 | 5040 | 60 | 2 | 12 |
| Nicolien M van der Kolk et al.2017 | 61 | CON | 0 | -0.80 | 0.722957 | 15 | 0 | 0 | 0 | 0 | 0 | 0 | 0 |
|  | 61 | AE | 250 | 1.10 | 0.319801 | 22 | 3.5 | 65 | 315 | 7560 | 30 | 3 | 24 |
| Daniele Volpe1 et al.2014 | 62 | BGT | 750 | -1.20 | 0.715788 | 17 | 3.8 | 10 | 760 | 6080 | 40 | 5 | 8 |
|  | 62 | AQE | 1000 | -2.10 | 0.798528 | 17 | 5.3 | 60 | 1060 | 8480 | 40 | 5 | 8 |
| Daniele Volpe1 et al.2016 | 63 | MulC | 1000 | -3.20 | 2.266956 | 11 | 4.75 | -50 | 950 | 7600 | 40 | 5 | 8 |
|  | 63 | AQE | 1000 | -1.40 | 0.569075 | 13 | 5.3 | 60 | 1060 | 8480 | 40 | 5 | 8 |
| Meg E. Morris et.al.2009 | 64 | MulC | 1000 | 0.10 | 0.763918 | 14 | 3.8 | 64 | 1064 | 2128 | 40 | 7 | 2 |
|  | 64 | SE | 1200 | -0.90 | 0.548374 | 14 | 4.3 | 4 | 1204 | 2408 | 40 | 7 | 2 |
| Helgerud J et al.2020 | 65 | CON | 0 | -0.50 | 0.916515 | 7 | 0 | 0 | 0 | 0 | 0 | 0 | 0 |
|  | 65 | RT | 1000 | -1.60 | 0.769848 | 15 | 5 | 125 | 1125 | 4500 | 45 | 5 | 4 |
| José Ma Cancela 2019 | 66 | MulC | 750 | 0.68 | 1.972689 | 5 | 4.75 | -37.5 | 712.5 | 2850 | 50 | 3 | 4 |
|  | 66 | AQE | 750 | 0.76 | 0.665861 | 7 | 5.3 | 45 | 795 | 3180 | 50 | 3 | 4 |
| Tamine T.C. Capato et al.2020 | 67 | CON | 0 | 0.50 | 2.243729 | 48 | 0 | 0 | 0 | 0 | 0 | 0 | 0 |
|  | 67 | BGT | 250 | -3.20 | 1.810173 | 50 | 3.8 | 92 | 342 | 1710 | 45 | 2 | 5 |
|  | 67 | SE | 250 | -6.90 | 1.882764 | 56 | 3.8 | 92 | 342 | 1710 | 45 | 2 | 5 |
| Tamine T.C.et al.2020 | 68 | BGT | 250 | 0.60 | 4.879369 | 18 | 3.8 | 92 | 342 | 1710 | 45 | 2 | 5 |
|  | 68 | SE | 250 | -6.20 | 3.361292 | 17 | 3.8 | 92 | 342 | 1710 | 45 | 2 | 5 |
| Gao et al.2014 | 69 | CON | 0 | 0.03 | 0.455584 | 39 | 0 | 0 | 0 | 0 | 0 | 0 | 0 |
|  | 69 | MBE | 500 | -1.37 | 0.481756 | 37 | 2.5 | -50 | 450 | 5400 | 60 | 3 | 12 |
| A Nieuwboer et al.2014 | 70 | CON | 0 | -0.30 | 0.89595 | 77 | 0 | 0 | 0 | 0 | 0 | 0 | 0 |
|  | 70 | SE | 250 | -0.40 | 0.774682 | 76 | 3 | 20 | 270 | 810 | 30 | 3 | 3 |
| Hsin- Hsuan Liu, PT et al.2022 | 71 | BGT | 500 | -2.64 | 2.188671 | 14 | 3.8 | -44 | 456 | 3648 | 60 | 2 | 8 |
|  | 71 | CON | 0 | -1.14 | 1.250511 | 14 | 0 | 0 | 0 | 0 | 0 | 0 | 0 |
| Zhenlan Li et al.2022 | 72 | MBE | 250 | -1.38 | 0.676598 | 20 | 3 | 110 | 360 | 4320 | 60 | 2 | 12 |
|  | 72 | CON | 0 | 1.95 | 0.943621 | 20 | 0 | 0 | 0 | 0 | 60 | 2 | 12 |
| Zhenlan Li et al.2022 | 73 | MBE | 500 | -1.5 | 0.676598 | 20 | 3 | 110 | 360 | 4320 | 60 | 2 | 12 |
|  | 73 | CON | 0 | 1.95 | 0.943621 | 20 | 0 | 0 | 0 | 0 | 60 | 2 | 12 |

## **List of included studies**

1. Tollár, J., Nagy, F., Kovács, N., & Hortobágyi, T. (2018). A high-intensity multicomponent agility intervention improves Parkinson patients’ clinical and motor symptoms. *Archives of physical medicine and rehabilitation*, *99*(12), 2478-2484.
2. Youm, C., Kim, Y., Noh, B., Lee, M., Kim, J., & Cheon, S. M. (2020). Impact of trunk resistance and stretching exercise on fall-related factors in patients with parkinson’s disease: A randomized controlled pilot study. *Sensors*, *20*(15), 4106
3. Vieira de Moraes Filho, A., Chaves, S. N., Martins, W. R., Tolentino, G. P., de Cássia Pereira Pinto Homem, R., Landim de Farias, G., ... & Jacó de Oliveira, R. (2020). Progressive resistance training improves bradykinesia, motor symptoms and functional performance in patients with Parkinson’s disease. *Clinical Interventions in Aging*, 87-95.
4. Capecci, M., Serpicelli, C., Fiorentini, L., Censi, G., Ferretti, M., Orni, C., ... & Ceravolo, M. G. (2014). Postural rehabilitation and Kinesio taping for axial postural disorders in Parkinson's disease. *Archives of physical medicine and rehabilitation*, *95*(6), 1067-1075.
5. Cherup, N. P., Strand, K. L., Lucchi, L., Wooten, S. V., Luca, C., & Signorile, J. F. (2021). Yoga meditation enhances proprioception and balance in individuals diagnosed with Parkinson’s disease. *Perceptual and motor skills*, *128*(1), 304-323.
6. Hackney, M. E., & Earhart, G. M. (2008). Tai Chi improves balance and mobility in people with Parkinson disease. *Gait & posture*, *28*(3), 456-460.
7. Khuzema, A., Brammatha, A., & Arul Selvan, V. (2020). Effect of home-based Tai Chi, Yoga or conventional balance exercise on functional balance and mobility among persons with idiopathic Parkinson's disease: An experimental study. *Hong Kong physiotherapy journal: official publication of the Hong Kong Physiotherapy Association Limited = Wu li chih liao*, *40*(1), 39–49. <https://doi.org/10.1142/S1013702520500055>
8. Kwok, J. Y. Y., Kwan, J. C. Y., Auyeung, M., Mok, V. C. T., Lau, C. K. Y., Choi, K. C., & Chan, H. Y. L. (2019). Effects of Mindfulness Yoga vs Stretching and Resistance Training Exercises on Anxiety and Depression for People With Parkinson Disease: A Randomized Clinical Trial. *JAMA neurology*, *76*(7), 755–763. <https://doi.org/10.1001/jamaneurol.2019.0534>
9. Liao, Y. Y., Yang, Y. R., Cheng, S. J., Wu, Y. R., Fuh, J. L., & Wang, R. Y. (2015). Virtual Reality-Based Training to Improve Obstacle-Crossing Performance and Dynamic Balance in Patients With Parkinson's Disease. *Neurorehabilitation and neural repair*, *29*(7), 658–667. <https://doi.org/10.1177/1545968314562111>
10. Liu, X. L., Chen, S., & Wang, Y. (2016). Effects of Health Qigong Exercises on Relieving Symptoms of Parkinson's Disease. *Evidence-based complementary and alternative medicine : eCAM*, *2016*, 5935782. <https://doi.org/10.1155/2016/5935782>
11. Schlenstedt, C., Paschen, S., Kruse, A., Raethjen, J., Weisser, B., & Deuschl, G. (2015). Resistance versus Balance Training to Improve Postural Control in Parkinson's Disease: A Randomized Rater Blinded Controlled Study. *PloS one*, *10*(10), e0140584. <https://doi.org/10.1371/journal.pone.0140584>
12. Santos, P., Machado, T., Santos, L., Ribeiro, N., & Melo, A. (2019). Efficacy of the Nintendo Wii combination with Conventional Exercises in the rehabilitation of individuals with Parkinson's disease: A randomized clinical trial. *NeuroRehabilitation*, *45*(2), 255–263. <https://doi.org/10.3233/NRE-192771>
13. Song, J., Paul, S. S., Caetano, M. J. D., Smith, S., Dibble, L. E., Love, R., Schoene, D., Menant, J. C., Sherrington, C., Lord, S. R., Canning, C. G., & Allen, N. E. (2018). Home-based step training using videogame technology in people with Parkinson's disease: a single-blinded randomised controlled trial. *Clinical rehabilitation*, *32*(3), 299–311. <https://doi.org/10.1177/0269215517721593>
14. Steib, S., Klamroth, S., Gaßner, H., Pasluosta, C., Eskofier, B., Winkler, J., Klucken, J., & Pfeifer, K. (2017). Perturbation During Treadmill Training Improves Dynamic Balance and Gait in Parkinson's Disease: A Single-Blind Randomized Controlled Pilot Trial. *Neurorehabilitation and neural repair*, *31*(8), 758–768. <https://doi.org/10.1177/1545968317721976>
15. Xiao, C. M., & Zhuang, Y. C. (2016). Effect of health Baduanjin Qigong for mild to moderate Parkinson's disease. *Geriatrics & gerontology international*, *16*(8), 911–919. <https://doi.org/10.1111/ggi.12571>
16. Xiao, C., Zhuang, Y., & Kang, Y. (2016). Effect of Health Qigong Baduanjin on Fall Prevention in Individuals with Parkinson's Disease. *Journal of the American Geriatrics Society*, *64*(11), e227–e228. <https://doi.org/10.1111/jgs.14438>
17. Yang, W. C., Wang, H. K., Wu, R. M., Lo, C. S., & Lin, K. H. (2016). Home-based virtual reality balance training and conventional balance training in Parkinson's disease: A randomized controlled trial. *Journal of the Formosan Medical Association = Taiwan yi zhi*, *115*(9), 734–743. <https://doi.org/10.1016/j.jfma.2015.07.012>
18. Yang, Y. R., Cheng, S. J., Lee, Y. J., Liu, Y. C., & Wang, R. Y. (2019). Cognitive and motor dual task gait training exerted specific training effects on dual task gait performance in individuals with Parkinson's disease: A randomized controlled pilot study. *PloS one*, *14*(6), e0218180. <https://doi.org/10.1371/journal.pone.0218180>
19. Zhang, T. Y., Hu, Y., Nie, Z. Y., Jin, R. X., Chen, F., Guan, Q., Hu, B., Gu, C. Y., Zhu, L., & Jin, L. J. (2015). Effects of Tai Chi and Multimodal Exercise Training on Movement and Balance Function in Mild to Moderate Idiopathic Parkinson Disease. *American journal of physical medicine & rehabilitation*, *94*(10 Suppl 1), 921–929. <https://doi.org/10.1097/PHM.0000000000000351>
20. Shih, M. C., Wang, R. Y., Cheng, S. J., & Yang, Y. R. (2016). Effects of a balance-based exergaming intervention using the Kinect sensor on posture stability in individuals with Parkinson's disease: a single-blinded randomized controlled trial. *Journal of neuroengineering and rehabilitation*, *13*(1), 78. <https://doi.org/10.1186/s12984-016-0185-y>
21. Calabrò, R. S., Naro, A., Filoni, S., Pullia, M., Billeri, L., Tomasello, P., Portaro, S., Di Lorenzo, G., Tomaino, C., & Bramanti, P. (2019). Walking to your right music: a randomized controlled trial on the novel use of treadmill plus music in Parkinson's disease. *Journal of neuroengineering and rehabilitation*, *16*(1), 68. <https://doi.org/10.1186/s12984-019-0533-9>
22. Demonceau, M., Maquet, D., Jidovtseff, B., Donneau, A. F., Bury, T., Croisier, J. L., Crielaard, J. M., Rodriguez de la Cruz, C., Delvaux, V., & Garraux, G. (2017). Effects of twelve weeks of aerobic or strength training in addition to standard care in Parkinson's disease: a controlled study. *European journal of physical and rehabilitation medicine*, *53*(2), 184–200. <https://doi.org/10.23736/S1973-9087.16.04272-6>
23. Vergara-Diaz, G., Osypiuk, K., Hausdorff, J. M., Bonato, P., Gow, B. J., Miranda, J. G., Sudarsky, L. R., Tarsy, D., Fox, M. D., Gardiner, P., Thomas, C. A., Macklin, E. A., & Wayne, P. M. (2018). Tai Chi for Reducing Dual-task Gait Variability, a Potential Mediator of Fall Risk in Parkinson's Disease: A Pilot Randomized Controlled Trial. *Global advances in health and medicine*, *7*, 2164956118775385. <https://doi.org/10.1177/2164956118775385>
24. Carvalho, A., Barbirato, D., Araujo, N., Martins, J. V., Cavalcanti, J. L., Santos, T. M., Coutinho, E. S., Laks, J., & Deslandes, A. C. (2015). Comparison of strength training, aerobic training, and additional physical therapy as supplementary treatments for Parkinson's disease: pilot study. *Clinical interventions in aging*, *10*, 183–191. <https://doi.org/10.2147/CIA.S68779>
25. Arcolin, I., Pisano, F., Delconte, C., Godi, M., Schieppati, M., Mezzani, A., Picco, D., Grasso, M., & Nardone, A. (2016). Intensive cycle ergometer training improves gait speed and endurance in patients with Parkinson's disease: A comparison with treadmill training. *Restorative neurology and neuroscience*, *34*(1), 125–138. <https://doi.org/10.3233/RNN-150506>
26. Arfa-Fatollahkhani, P., Safar Cherati, A., Habibi, S. A. H., Shahidi, G. A., Sohrabi, A., & Zamani, B. (2019). Effects of treadmill training on the balance, functional capacity and quality of life in Parkinson's disease: A randomized clinical trial. *Journal of complementary & integrative medicine*, *17*(1), /j/jcim.2019.17.issue-1/jcim-2018-0245/jcim-2018-0245.xml. <https://doi.org/10.1515/jcim-2018-0245>
27. Bang, D. H., & Shin, W. S. (2017). Effects of an intensive Nordic walking intervention on the balance function and walking ability of individuals with Parkinson's disease: a randomized controlled pilot trial. *Aging clinical and experimental research*, *29*(5), 993–999. <https://doi.org/10.1007/s40520-016-0648-9>
28. Bello, O., Sanchez, J. A., Lopez-Alonso, V., Márquez, G., Morenilla, L., Castro, X., Giraldez, M., Santos-García, D., & Fernandez-del-Olmo, M. (2013). The effects of treadmill or overground walking training program on gait in Parkinson's disease. *Gait & posture*, *38*(4), 590–595. <https://doi.org/10.1016/j.gaitpost.2013.02.005>
29. Ebersbach, G., Ebersbach, A., Edler, D., Kaufhold, O., Kusch, M., Kupsch, A., & Wissel, J. (2010). Comparing exercise in Parkinson's disease--the Berlin LSVT®BIG study. *Movement disorders : official journal of the Movement Disorder Society*, *25*(12), 1902–1908. <https://doi.org/10.1002/mds.23212>
30. Carpinella, I., Cattaneo, D., Bonora, G., Bowman, T., Martina, L., Montesano, A., & Ferrarin, M. (2017). Wearable Sensor-Based Biofeedback Training for Balance and Gait in Parkinson Disease: A Pilot Randomized Controlled Trial. *Archives of physical medicine and rehabilitation*, *98*(4), 622–630.e3. <https://doi.org/10.1016/j.apmr.2016.11.003>
31. Cheng, F. Y., Yang, Y. R., Wu, Y. R., Cheng, S. J., & Wang, R. Y. (2017). Effects of curved-walking training on curved-walking performance and freezing of gait in individuals with Parkinson's disease: A randomized controlled trial. *Parkinsonism & related disorders*, *43*, 20–26. <https://doi.org/10.1016/j.parkreldis.2017.06.021>
32. Rios Romenets, S., Anang, J., Fereshtehnejad, S. M., Pelletier, A., & Postuma, R. (2015). Tango for treatment of motor and non-motor manifestations in Parkinson's disease: a randomized control study. *Complementary therapies in medicine*, *23*(2), 175–184. <https://doi.org/10.1016/j.ctim.2015.01.015>
33. Costa-Ribeiro, A., Maux, A., Bosford, T., Aoki, Y., Castro, R., Baltar, A., Shirahige, L., Moura Filho, A., Nitsche, M. A., & Monte-Silva, K. (2017). Transcranial direct current stimulation associated with gait training in Parkinson's disease: A pilot randomized clinical trial. *Developmental neurorehabilitation*, *20*(3), 121–128. <https://doi.org/10.3109/17518423.2015.1131755>
34. Cugusi, L., Solla, P., Serpe, R., Carzedda, T., Piras, L., Oggianu, M., Gabba, S., Di Blasio, A., Bergamin, M., Cannas, A., Marrosu, F., & Mercuro, G. (2015). Effects of a Nordic Walking program on motor and non-motor symptoms, functional performance and body composition in patients with Parkinson's disease. *NeuroRehabilitation*, *37*(2), 245–254. <https://doi.org/10.3233/NRE-151257>
35. Schlenstedt, C., Paschen, S., Kruse, A., Raethjen, J., Weisser, B., & Deuschl, G. (2015). Resistance versus Balance Training to Improve Postural Control in Parkinson's Disease: A Randomized Rater Blinded Controlled Study. *PloS one*, *10*(10), e0140584. <https://doi.org/10.1371/journal.pone.0140584>
36. Frazzitta, G., Bossio, F., Maestri, R., Palamara, G., Bera, R., & Ferrazzoli, D. (2015). Crossover versus Stabilometric Platform for the Treatment of Balance Dysfunction in Parkinson's Disease: A Randomized Study. *BioMed research international*, *2015*, 878472. <https://doi.org/10.1155/2015/878472>
37. Goodwin, V. A., Richards, S. H., Henley, W., Ewings, P., Taylor, A. H., & Campbell, J. L. (2011). An exercise intervention to prevent falls in people with Parkinson's disease: a pragmatic randomised controlled trial. *Journal of neurology, neurosurgery, and psychiatry*, *82*(11), 1232–1238. <https://doi.org/10.1136/jnnp-2011-300919>
38. Hackney, M. E., Kantorovich, S., Levin, R., & Earhart, G. M. (2007). Effects of tango on functional mobility in Parkinson's disease: a preliminary study. *Journal of neurologic physical therapy: JNPT*, *31*(4), 173–179. <https://doi.org/10.1097/NPT.0b013e31815ce78b>
39. Hubble, R. P., Naughton, G., Silburn, P. A., & Cole, M. H. (2018). Trunk Exercises Improve Gait Symmetry in Parkinson Disease: A Blind Phase II Randomized Controlled Trial. *American journal of physical medicine & rehabilitation*, *97*(3), 151–159. <https://doi.org/10.1097/PHM.0000000000000858>
40. Silva, A. Z. D., & Israel, V. L. (2019). Effects of dual-task aquatic exercises on functional mobility, balance and gait of individuals with Parkinson's disease: A randomized clinical trial with a 3-month follow-up. *Complementary therapies in medicine*, *42*, 119–124. <https://doi.org/10.1016/j.ctim.2018.10.023>
41. Volpe, D., Giantin, M. G., & Fasano, A. (2014). A wearable proprioceptive stabilizer (Equistasi®) for rehabilitation of postural instability in Parkinson's disease: a phase II randomized double-blind, double-dummy, controlled study. *PloS one*, *9*(11), e112065. <https://doi.org/10.1371/journal.pone.0112065>
42. Sage, M. D., & Almeida, Q. J. (2009). Symptom and gait changes after sensory attention focused exercise vs aerobic training in Parkinson's disease. *Movement disorders : official journal of the Movement Disorder Society*, *24*(8), 1132–1138. <https://doi.org/10.1002/mds.22469>
43. Kadivar, Z., Corcos, D. M., Foto, J., & Hondzinski, J. M. (2011). Effect of step training and rhythmic auditory stimulation on functional performance in Parkinson patients. *Neurorehabilitation and neural repair*, *25*(7), 626–635. <https://doi.org/10.1177/1545968311401627>
44. Solla, P., Cugusi, L., Bertoli, M., Cereatti, A., Della Croce, U., Pani, D., Fadda, L., Cannas, A., Marrosu, F., Defazio, G., & Mercuro, G. (2019). Sardinian Folk Dance for Individuals with Parkinson's Disease: A Randomized Controlled Pilot Trial. *Journal of alternative and complementary medicine (New York, N.Y.)*, *25*(3), 305–316. <https://doi.org/10.1089/acm.2018.0413>
45. Michels, K., Dubaz, O., Hornthal, E., & Bega, D. (2018). "Dance Therapy" as a psychotherapeutic movement intervention in Parkinson's disease. *Complementary therapies in medicine*, *40*, 248–252. <https://doi.org/10.1016/j.ctim.2018.07.005>
46. Kunkel, D., Fitton, C., Roberts, L., Pickering, R. M., Roberts, H. C., Wiles, R., Hulbert, S., Robison, J., & Ashburn, A. (2017). A randomized controlled feasibility trial exploring partnered ballroom dancing for people with Parkinson's disease. *Clinical rehabilitation*, *31*(10), 1340–1350. <https://doi.org/10.1177/0269215517694930>
47. Shulman, L. M., Katzel, L. I., Ivey, F. M., Sorkin, J. D., Favors, K., Anderson, K. E., Smith, B. A., Reich, S. G., Weiner, W. J., & Macko, R. F. (2013). Randomized clinical trial of 3 types of physical exercise for patients with Parkinson disease. *JAMA neurology*, *70*(2), 183–190. <https://doi.org/10.1001/jamaneurol.2013.646>
48. Bekkers, E. M. J., Mirelman, A., Alcock, L., Rochester, L., Nieuwhof, F., Bloem, B. R., Pelosin, E., Avanzino, L., Cereatti, A., Della Croce, U., Hausdorff, J. M., & Nieuwboer, A. (2020). Do Patients With Parkinson's Disease With Freezing of Gait Respond Differently Than Those Without to Treadmill Training Augmented by Virtual Reality?. *Neurorehabilitation and neural repair*, *34*(5), 440–449. <https://doi.org/10.1177/1545968320912756>
49. Pohl, P., Dizdar, N., & Hallert, E. (2013). The Ronnie Gardiner Rhythm and Music Method - a feasibility study in Parkinson's disease. *Disability and rehabilitation*, *35*(26), 2197–2204. <https://doi.org/10.3109/09638288.2013.774060>
50. Vergara-Diaz, G., Osypiuk, K., Hausdorff, J. M., Bonato, P., Gow, B. J., Miranda, J. G., Sudarsky, L. R., Tarsy, D., Fox, M. D., Gardiner, P., Thomas, C. A., Macklin, E. A., & Wayne, P. M. (2018). Tai Chi for Reducing Dual-task Gait Variability, a Potential Mediator of Fall Risk in Parkinson's Disease: A Pilot Randomized Controlled Trial. *Global advances in health and medicine*, *7*, 2164956118775385. <https://doi.org/10.1177/2164956118775385>
51. Kurt, E. E., Büyükturan, B., Büyükturan, Ö., Erdem, H. R., & Tuncay, F. (2018). Effects of Ai Chi on balance, quality of life, functional mobility, and motor impairment in patients with Parkinson's disease. Disability and rehabilitation, 40(7), 791–797. <https://doi.org/10.1080/09638288.2016.1276972>
52. Kurtais, Y., Kutlay, S., Tur, B. S., Gok, H., & Akbostanci, C. (2008). Does treadmill training improve lower-extremity tasks in Parkinson disease? A randomized controlled trial. *Clinical journal of sport medicine : official journal of the Canadian Academy of Sport Medicine*, *18*(3), 289–291. <https://doi.org/10.1097/JSM.0b013e318170626d>
53. Leal, L. C., Abrahin, O., Rodrigues, R. P., da Silva, M. C., Araújo, A. P., de Sousa, E. C., Pimentel, C. P., & Cortinhas-Alves, E. A. (2019). Low-volume resistance training improves the functional capacity of older individuals with Parkinson's disease. *Geriatrics & gerontology international*, *19*(7), 635–640. <https://doi.org/10.1111/ggi.13682>
54. Monteiro, E. P., Franzoni, L. T., Cubillos, D. M., de Oliveira Fagundes, A., Carvalho, A. R., Oliveira, H. B., Pantoja, P. D., Schuch, F. B., Rieder, C. R., Martinez, F. G., & Peyré-Tartaruga, L. A. (2017). Effects of Nordic walking training on functional parameters in Parkinson's disease: a randomized controlled clinical trial. *Scandinavian journal of medicine & science in sports*, *27*(3), 351–358. <https://doi.org/10.1111/sms.12652>
55. Morris, M. E., Menz, H. B., McGinley, J. L., Watts, J. J., Huxham, F. E., Murphy, A. T., Danoudis, M. E., & Iansek, R. (2015). A Randomized Controlled Trial to Reduce Falls in People With Parkinson's Disease. *Neurorehabilitation and neural repair*, *29*(8), 777–785. <https://doi.org/10.1177/1545968314565511>
56. Palamara, G., Gotti, F., Maestri, R., Bera, R., Gargantini, R., Bossio, F., Zivi, I., Volpe, D., Ferrazzoli, D., & Frazzitta, G. (2017). Land Plus Aquatic Therapy Versus Land-Based Rehabilitation Alone for the Treatment of Balance Dysfunction in Parkinson Disease: A Randomized Controlled Study With 6-Month Follow-Up. *Archives of physical medicine and rehabilitation*, *98*(6), 1077–1085. <https://doi.org/10.1016/j.apmr.2017.01.025>
57. Park, A., Zid, D., Russell, J., Malone, A., Rendon, A., Wehr, A., & Li, X. (2014). Effects of a formal exercise program on Parkinson's disease: a pilot study using a delayed start design. *Parkinsonism & related disorders*, *20*(1), 106–111. <https://doi.org/10.1016/j.parkreldis.2013.10.003>
58. Poliakoff, E., Galpin, A. J., McDonald, K., Kellett, M., Dick, J. P., Hayes, S., & Wearden, A. J. (2013). The effect of gym training on multiple outcomes in Parkinson's disease: a pilot randomised waiting-list controlled trial. *NeuroRehabilitation*, *32*(1), 125–134. <https://doi.org/10.3233/NRE-130829>
59. Schlick, C., Ernst, A., Bötzel, K., Plate, A., Pelykh, O., & Ilmberger, J. (2016). Visual cues combined with treadmill training to improve gait performance in Parkinson's disease: a pilot randomized controlled trial. *Clinical rehabilitation*, *30*(5), 463–471. <https://doi.org/10.1177/0269215515588836>
60. Silva-Batista, C., Corcos, D. M., Barroso, R., David, F. J., Kanegusuku, H., Forjaz, C., DE Mello, M. T., Roschel, H., Tricoli, V., & Ugrinowitsch, C. (2017). Instability Resistance Training Improves Neuromuscular Outcome in Parkinson's Disease. *Medicine and science in sports and exercise*, *49*(4), 652–660. <https://doi.org/10.1249/MSS.0000000000001159>
61. Silva-Batista, C., Corcos, D. M., Roschel, H., Kanegusuku, H., Gobbi, L. T., Piemonte, M. E., Mattos, E. C., DE Mello, M. T., Forjaz, C. L., Tricoli, V., & Ugrinowitsch, C. (2016). Resistance Training with Instability for Patients with Parkinson's Disease. *Medicine and science in sports and exercise*, *48*(9), 1678–1687. <https://doi.org/10.1249/MSS.0000000000000945>
62. van der Kolk, N. M., de Vries, N. M., Penko, A. L., van der Vlugt, M., Mulder, A. A., Post, B., Alberts, J. L., & Bloem, B. R. (2018). A remotely supervised home-based aerobic exercise programme is feasible for patients with Parkinson's disease: results of a small randomised feasibility trial. *Journal of neurology, neurosurgery, and psychiatry*, *89*(9), 1003–1005. <https://doi.org/10.1136/jnnp-2017-315728>
63. Volpe, D., Giantin, M. G., Maestri, R., & Frazzitta, G. (2014). Comparing the effects of hydrotherapy and land-based therapy on balance in patients with Parkinson's disease: a randomized controlled pilot study. *Clinical rehabilitation*, *28*(12), 1210–1217. <https://doi.org/10.1177/0269215514536060>
64. Volpe, D., Giantin, M. G., Manuela, P., Filippetto, C., Pelosin, E., Abbruzzese, G., & Antonini, A. (2017). Water-based vs. non-water-based physiotherapy for rehabilitation of postural deformities in Parkinson's disease: a randomized controlled pilot study. *Clinical rehabilitation*, *31*(8), 1107–1115. <https://doi.org/10.1177/0269215516664122>
65. Morris, M. E., Iansek, R., & Kirkwood, B. (2009). A randomized controlled trial of movement strategies compared with exercise for people with Parkinson's disease. *Movement disorders : official journal of the Movement Disorder Society*, *24*(1), 64–71. <https://doi.org/10.1002/mds.22295>
66. Helgerud, J., Thomsen, S. N., Hoff, J., Strandbråten, A., Leivseth, G., Unhjem, R., & Wang, E. (2020). Maximal strength training in patients with Parkinson's disease: impact on efferent neural drive, force-generating capacity, and functional performance. *Journal of applied physiology (Bethesda, Md.: 1985)*, *129*(4), 683–690. <https://doi.org/10.1152/japplphysiol.00208.2020>
67. Cancela, J. M., Mollinedo, I., Montalvo, S., & Vila Suárez, M. E. (2020). Effects of a High-Intensity Progressive-Cycle Program on Quality of Life and Motor Symptomatology in a Parkinson's Disease Population: A Pilot Randomized Controlled Trial. *Rejuvenation research*, *23*(6), 508–515. <https://doi.org/10.1089/rej.2019.2267>
68. Capato, T. T. C., de Vries, N. M., IntHout, J., Barbosa, E. R., Nonnekes, J., & Bloem, B. R. (2020). Multimodal Balance Training Supported by Rhythmical Auditory Stimuli in Parkinson's Disease: A Randomized Clinical Trial. *Journal of Parkinson's disease*, *10*(1), 333–346. <https://doi.org/10.3233/JPD-191752>
69. Capato, T. T. C., Nonnekes, J., de Vries, N. M., IntHout, J., Barbosa, E. R., & Bloem, B. R. (2020). Effects of multimodal balance training supported by rhythmical auditory stimuli in people with advanced stages of Parkinson's disease: a pilot randomized clinical trial. *Journal of the neurological sciences*, *418*, 117086. <https://doi.org/10.1016/j.jns.2020.117086>
70. Gao, Q., Leung, A., Yang, Y., Wei, Q., Guan, M., Jia, C., & He, C. (2014). Effects of Tai Chi on balance and fall prevention in Parkinson's disease: a randomized controlled trial. *Clinical rehabilitation*, *28*(8), 748–753. <https://doi.org/10.1177/0269215514521044>
71. Nieuwboer, A., Kwakkel, G., Rochester, L., Jones, D., van Wegen, E., Willems, A. M., Chavret, F., Hetherington, V., Baker, K., & Lim, I. (2007). Cueing training in the home improves gait-related mobility in Parkinson's disease: the RESCUE trial. *Journal of neurology, neurosurgery, and psychiatry*, *78*(2), 134–140. <https://doi.org/10.1136/jnnp.200X.097923>
72. Liu, H. H., Wang, R. Y., Cheng, S. J., Liao, K. K., Zhou, J. H., & Yang, Y. R. (2022). Balance Training Modulates Cortical Inhibition in Individuals with Parkinson's Disease: A Randomized Controlled Trial. *Neurorehabilitation and neural repair*, *36*(9), 613–620. <https://doi.org/10.1177/15459683221119761>
73. Li, Z., Wang, T., Shen, M., Song, T., He, J., Guo, W., Wang, Z., & Zhuang, J. (2022). Comparison of Wuqinxi Qigong with Stretching on Single- and Dual-Task Gait, Motor Symptoms and Quality of Life in Parkinson's Disease: A Preliminary Randomized Control Study. *International journal of environmental research and public health*, *19*(13), 8042. <https://doi.org/10.3390/ijerph19138042>

# **Supplementary File 4: Assessment of** **Model Consistency**

We analyzed the data with the consistency model and the unrelated mean effect model, and compared the differences in the deviation, the number of estimated parameters in the network, and the Deviance Informative Criterion (DIC) indicators of the two models. If these are similar, it means that our research has good consistency (Wheeler et al., 2010). Comparison of these parameters indicated good consistency across models (Table 4.1).

## **Table 4.1. Consistent and UME models fit comparison**

| **Model** | **pD** | **Residual deviance** | **Deviance** | **DIC** | **SD** |
| --- | --- | --- | --- | --- | --- |
| Consistent | 141.6 | 161.287 | 377.245 | 518.3 | 1.299 |
| UME | 139.8 | 167.196 | 383.154 | 522.5 | 1.360 |

pD: Number of estimated parameters; DIC: Deviance Informative Criterion; SD: Standard Deviation; UME: Unrelated Mean Effects. Scientific literature indicated that the main indicator to assess the model fit is the DIC. As lower DIC, better fit.

# **Supplementary File 5: Node-splitting analysis** **of** **inconsistency**

We assessed inconsistency via MBNMA node-splitting approach. This method splits and compares contributions for a particular treatment contrast into direct and indirect evidence (van Valkenhoef et al., 2016). Similar effects denote good consistency. Table 5.1 below present the results for node-splitting analysis of inconsistency in this meta-analysis. The results showed that there was no inconsistency in our study (P>0.05).

## **Table 5.1 Node-splitting analysis of inconsistency**

| **Comparison** |  | **p-value** | **Median** | **2.50%** | **97.50%** |
| --- | --- | --- | --- | --- | --- |
| TT_250 vs SE_500 |  | 0.511 |  |  |  |
| -> direct |  |  | 1.361 | -2.056 | 4.682 |
| -> indirect |  |  | 0.847 | -0.343 | 2.026 |
| -> MBNMA |  |  | 0.958 | -0.182 | 2.093 |
|  |  |  |  |  |  |
| TT_250 vs SE_250 |  | 0.802 |  |  |  |
| -> direct |  |  | 0.975 | -0.767 | 2.708 |
| -> indirect |  |  | 0.613 | -0.801 | 2.024 |
| -> MBNMA |  |  | 0.77 | -0.353 | 1.844 |
|  |  |  |  |  |  |
| SE_500 vs SE_250 |  | 0.12 |  |  |  |
| -> direct |  |  | 0.714 | -3.078 | 4.314 |
| -> indirect |  |  | -0.17 | -0.496 | -0.009 |
| -> MBNMA |  |  | -0.166 | -0.47 | -0.01 |
|  |  |  |  |  |  |
| TT_750 vs RT_500 |  | 0.531 |  |  |  |
| -> direct |  |  | -0.044 | -2.812 | 2.697 |
| -> indirect |  |  | 1.193 | -0.459 | 2.715 |
| -> MBNMA |  |  | 0.915 | -0.546 | 2.285 |
|  |  |  |  |  |  |
| SE_1200 vs MulC_1000 |  | 0.507 |  |  |  |
| -> direct |  |  | -1.004 | -4.267 | 2.182 |
| -> indirect |  |  | 0.197 | -1.197 | 1.652 |
| -> MBNMA |  |  | 0.34 | -0.908 | 1.623 |
|  |  |  |  |  |  |
| RT_750 vs MBE_750 |  | 0.435 |  |  |  |
| -> direct |  |  | 1.171 | -1.82 | 4.147 |
| -> indirect |  |  | -0.307 | -1.605 | 1.003 |
| -> MBNMA |  |  | -0.431 | -1.678 | 0.786 |
|  |  |  |  |  |  |
| MulC_500 vs MBE_250 |  | 0.628 |  |  |  |
| -> direct |  |  | -0.252 | -3.238 | 2.873 |
| -> indirect |  |  | -0.628 | -1.998 | 0.705 |
| -> MBNMA |  |  | -0.463 | -1.652 | 0.756 |
|  |  |  |  |  |  |
| RT_500 vs Dance_250 |  | 0.355 |  |  |  |
| -> direct |  |  | -0.751 | -3.614 | 1.947 |
| -> indirect |  |  | 1.261 | -0.352 | 2.896 |
| -> MBNMA |  |  | 1.109 | -0.225 | 2.569 |
|  |  |  |  |  |  |
| SE_750 vs BGT_750 |  | 0.404 |  |  |  |
| -> direct |  |  | -1.293 | -4.688 | 2.15 |
| -> indirect |  |  | 0.347 | -1.013 | 1.739 |
| -> MBNMA |  |  | 0.513 | -0.726 | 1.775 |
|  |  |  |  |  |  |
| SE_500 vs BGT_500 |  | 0.533 |  |  |  |
| -> direct |  |  | 0.074 | -3.408 | 3.556 |
| -> indirect |  |  | 0.529 | -0.678 | 1.795 |
| -> MBNMA |  |  | 0.495 | -0.661 | 1.718 |
|  |  |  |  |  |  |
| SE_250 vs BGT_500 |  | 0.17 |  |  |  |
| -> direct |  |  | -3.102 | -6.415 | 0.157 |
| -> indirect |  |  | -0.122 | -1.295 | 1.105 |
| -> MBNMA |  |  | 0.303 | -0.835 | 1.493 |
|  |  |  |  |  |  |
| SE_250 vs BGT_250 |  | 0.118 |  |  |  |
| -> direct |  |  | -3.143 | -6.163 | -0.205 |
| -> indirect |  |  | 0.075 | -1.075 | 1.249 |
| -> MBNMA |  |  | 0.458 | -0.593 | 1.541 |
|  |  |  |  |  |  |
| RT_500 vs BGT_250 |  | 0.29 |  |  |  |
| -> direct |  |  | -1.536 | -4.593 | 1.597 |
| -> indirect |  |  | 0.575 | -0.659 | 1.795 |
| -> MBNMA |  |  | 0.72 | -0.398 | 1.901 |
|  |  |  |  |  |  |
| MBE_250 vs BGT_250 |  | 0.515 |  |  |  |
| -> direct |  |  | 0.069 | -3.317 | 3.441 |
| -> indirect |  |  | 0.942 | -0.355 | 2.345 |
| -> MBNMA |  |  | 0.783 | -0.418 | 2.023 |
|  |  |  |  |  |  |
| MulC_1000 vs AQE_1500 | | 0.138 |  |  |  |
| -> direct |  |  | 4.168 | -0.387 | 8.816 |
| -> indirect |  |  | -0.331 | -2.031 | 1.368 |
| -> MBNMA |  |  | -0.805 | -2.395 | 0.758 |
|  |  |  |  |  |  |
| MulC_1000 vs AQE_1200 | | 0.463 |  |  |  |
| -> direct |  |  | 1.019 | -2.083 | 4.137 |
| -> indirect |  |  | -0.693 | -2.492 | 1.191 |
| -> MBNMA |  |  | -0.775 | -2.34 | 0.765 |
|  |  |  |  |  |  |
| MulC_1000 vs AQE_1000 | | 0.463 |  |  |  |
| -> direct |  |  | -1.935 | -7.05 | 3.298 |
| -> indirect |  |  | -0.957 | -2.572 | 0.658 |
| -> MBNMA |  |  | -0.741 | -2.298 | 0.77 |
|  |  |  |  |  |  |
| BGT_750 vs AQE_1000 |  | 0.502 |  |  |  |
| -> direct |  |  | 0.813 | -2.387 | 4.076 |
| -> indirect |  |  | -0.959 | -3.084 | 1.125 |
| -> MBNMA |  |  | -0.982 | -2.676 | 0.773 |
|  |  |  |  |  |  |
| MulC_750 vs AQE_750 |  | 0.504 |  |  |  |
| -> direct |  |  | -0.202 | -4.773 | 4.663 |
| -> indirect |  |  | -0.806 | -2.397 | 0.827 |
| -> MBNMA |  |  | -0.725 | -2.227 | 0.747 |
|  |  |  |  |  |  |
| TT_500 vs AE_750 |  | 0.673 |  |  |  |
| -> direct |  |  | 0.077 | -1.945 | 2.059 |
| -> indirect |  |  | -0.692 | -2.406 | 1.036 |
| -> MBNMA |  |  | -0.33 | -1.649 | 0.956 |
|  |  |  |  |  |  |
| AQE_500 vs AE_750 |  | 0.416 |  |  |  |
| -> direct |  |  | 0.172 | -2.899 | 3.287 |
| -> indirect |  |  | 2.175 | 0.282 | 3.99 |
| -> MBNMA |  |  | 1.572 | -0.053 | 3.092 |
|  |  |  |  |  |  |
| BGT_500 vs AE_500 |  | 0.04 |  |  |  |
| -> direct |  |  | -4.225 | -6.679 | -1.796 |
| -> indirect |  |  | -0.243 | -1.577 | 1.061 |
| -> MBNMA |  |  | 0.729 | -0.555 | 1.953 |
|  |  |  |  |  |  |
| RT_250 vs AE_250 |  | 0.397 |  |  |  |
| -> direct |  |  | 0.031 | -3.346 | 3.425 |
| -> indirect |  |  | 1.28 | 0.25 | 2.311 |
| -> MBNMA |  |  | 1.189 | 0.2 | 2.176 |
|  |  |  |  |  |  |
| BGT_250 vs AE_250 |  | 0.503 |  |  |  |
| -> direct |  |  | 0.444 | -3.469 | 4.13 |
| -> indirect |  |  | 0.773 | -0.448 | 1.965 |
| -> MBNMA |  |  | 0.632 | -0.525 | 1.738 |
|  |  |  |  |  |  |
| RT_1200 vs Placebo_0 |  | 0.154 |  |  |  |
| -> direct |  |  | -0.086 | -2.276 | 1.995 |
| -> indirect |  |  | -2.108 | -2.997 | -1.324 |
| -> MBNMA |  |  | -1.883 | -2.708 | -1.058 |
|  |  |  |  |  |  |
| RT_1000 vs Placebo_0 |  | 0.379 |  |  |  |
| -> direct |  |  | -1.148 | -4.475 | 2.372 |
| -> indirect |  |  | -1.885 | -2.714 | -1.114 |
| -> MBNMA |  |  | -1.863 | -2.686 | -1.055 |
|  |  |  |  |  |  |
| MulC_250 vs Placebo_0 |  | 0.503 |  |  |  |
| -> direct |  |  | -0.615 | -3.264 | 1.915 |
| -> indirect |  |  | -1.275 | -2.343 | -0.357 |
| -> MBNMA |  |  | -1.197 | -2.085 | -0.318 |
|  |  |  |  |  |  |
| Dance_750 vs Placebo_0 | | 0.592 |  |  |  |
| -> direct |  |  | 0.128 | -2.846 | 3.088 |
| -> indirect |  |  | -1.072 | -2.934 | 0.742 |
| -> MBNMA |  |  | -0.768 | -2.292 | 0.799 |
|  |  |  |  |  |  |
| Dance_500 vs Placebo_0 | | 0.644 |  |  |  |
| -> direct |  |  | -1.332 | -3.886 | 1.32 |
| -> indirect |  |  | -0.396 | -2.14 | 1.264 |
| -> MBNMA |  |  | -0.736 | -2.184 | 0.766 |

The number after the exercise type represents the weekly dose (MET-min). AE Aerobic Exercise, AQE Aquatic Exercise, BGT Balance and Gait Training,CON Control group, MulC Multicomponent Exercise Program, RT Resistance Training, TT Treadmil traing, TC Tai Chi, SE Sensory Exercise.

# **Supplementary File 6: Non-linear functions and models fit comparison**

The different doses of exercise treatments were meta-analysed as independent and unrelated treatments (i.e., “split” NMA). This step is useful to determine which function fits the data better and should subsequently be used in a Model-Based Network Meta-Analysis (MBNMA) (Pedder, 2021). Figure 6.1 show the different responses (MD) of each dose for different treatments, respectively.

**Figure 6.1** “Split” NMA of different exercise treatment agents. AE Aerobic Exercise, AQE Aquatic Exercise, BGT Balance and Gait Training,CON Control group, MulC Multicomponent Exercise Program, RT Resistance Training, TT Treadmil traing, TC Tai Chi, SE Sensory Exercise.

Table 6.1 shows the fit indices from each of the models fitted. For our data, restricted cubic splines show the best fit and were therefore used in subsequent analyses.

## **Table 6.1** Models fit comparison

| **Model** | **DIC** | **SD** | **Deviance** | **Residual deviance** | **pD** |
| --- | --- | --- | --- | --- | --- |
| Emax  (common treatment effects) | 647.3 | NA | 558.046 | 342.089 | 89.6 |
| Emax  (random treatment effects) | 507.9 | 1.244 | 378.028 | 162.070 | 130.5 |
| Exponential  (common treatment effects) | 654.4 | NA | 566.164 | 350.207 | 88.9 |
| Exponential  (random treatment effects) | 506.3 | 1.251 | 377.321 | 161.364 | 129.9 |
| Restricted cubic spline  (common treatment effects; 3 knots) | 569.7 | NA | 472.276 | 256.318 | 98.1 |
| Restricted cubic spline  (random treatment effects; 3 knots) | 499.3 | 1.139 | 368.067 | 159.110 | 126.3 |
| Non-parametric monotonically up (common treatment effects) | 803.5 | NA | 716.452 | 500.495 | 87.7 |
| Non-parametric monotonically up (random treatment effects) | 530.9 | 2.623 | 382.635 | 166.677 | 149.1 |
| Linear (common treatment effects) | 645.8 | NA | 557.344 | 341.387 | 89.1 |
| Linear (random treatment effects) | 510.3 | 1.315 | 378.382 | 162.424 | 132.4 |

DIC = Deviance Information Criterion; SD = Between-study Standard Deviation; pD: Number of estimated parameters; NA = Not Applicable. The SD is presented as the main value and (95% Credible Intervals).

Further to model fit indices, deviance plots showing the contribution of each data point to the residual deviance are also useful to confirm the robustness of model selection (Pedder, 2021). Each data point should contribute about 1 to the posterior mean deviance, which indicates good model fit (Dias et al., 2013). The deviance plot for treatment effects confirm the robustness of our model selection (Figure 6.2).

Figure 6.2. Deviance plots at treatment-level. AE Aerobic Exercise, AQE Aquatic Exercise, BGT Balance and Gait Training,CON Control group, MulC Multicomponent Exercise Program, RT Resistance Training, TT Treadmil traing, TC Tai Chi, SE Sensory Exercise.

# **Supplementary File 7:** **Study-level risk of bias analysis**

**
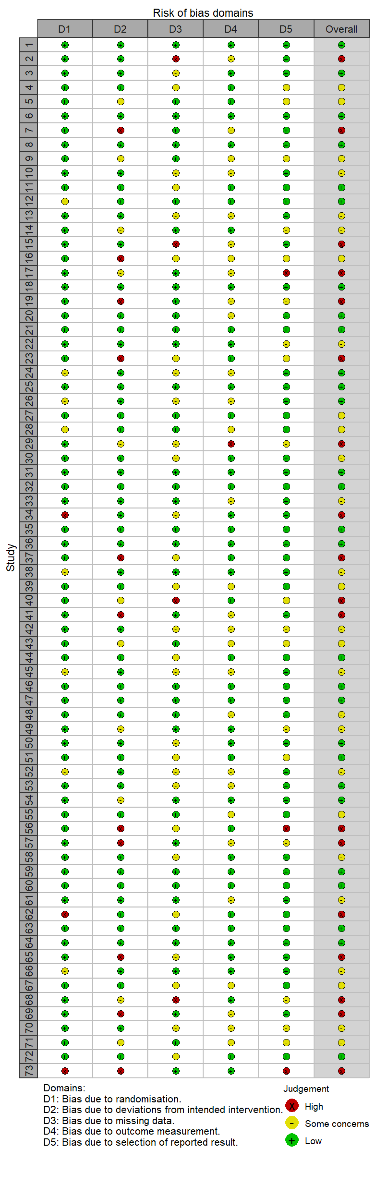
**

**Reference**

Dias, S., Sutton, A. J., Ades, A. E., & Welton, N. J. (2013). Evidence synthesis for decision making 2: a generalized linear modeling framework for pairwise and network meta-analysis of randomized controlled trials. *Medical Decision Making : an International Journal of the Society For Medical Decision Making, 33*(5), 607-617. doi:10.1177/0272989X12458724

Pedder, H. (2021). *MBNMAdose: An R package for incorporating dose-response information into Network Meta-Analysis.* Paper presented at the Evidence Synthesis and Meta-Analysis in R Conference 2021.

van Valkenhoef, G., Dias, S., Ades, A. E., & Welton, N. J. (2016). Automated generation of node-splitting models for assessment of inconsistency in network meta-analysis. *Research Synthesis Methods, 7*(1), 80-93. doi:10.1002/jrsm.1167

Wheeler, D. C., Hickson, D. A., & Waller, L. A. (2010). Assessing Local Model Adequacy in Bayesian Hierarchical Models Using the Partitioned Deviance Information Criterion. *Computational Statistics & Data Analysis, 54*(6), 1657-1671. Retrieved from <https://pubmed.ncbi.nlm.nih.gov/21243121>
